# The sentinel against brain injury post-subarachnoid hemorrhage: efferocytosis of erythrocytes by leptomeningeal lymphatic endothelial cells

**DOI:** 10.7150/thno.103701

**Published:** 2025-01-20

**Authors:** Hong-Ji Deng, Yun-Huo Xu, Kun Wu, Yun-Cong Li, Yong-Jin Zhang, Han-Fu Yu, Chong Li, Dan Xu, Fei Wang

**Affiliations:** 1 Department of Neurosurgery, The First Affiliated Hospital of Kunming Medical University, Kunming, China.; 2 Department of Clinical Laboratory, The First Affiliated Hospital of Kunming Medical University, Kunming, China.; 3 Clinical Medical Research Center, The First Affiliated Hospital of Kunming Medical University, Kunming, China.; 4 Department of Dermatology, The First Affiliated Hospital of Kunming Medical University, Kunming, China.

**Keywords:** subarachnoid hemorrhage, leptomeningeal lymphatic endothelial cells, efferocytosis, NHLRC2, neuroprotection

## Abstract

**Rationale:** The clearance of extravasated erythrocytes represents the most reasonable strategy against brain injury post-subarachnoid hemorrhage (SAH). There is little knowledge about the autologous clearance of extravasated erythrocytes post-SAH. The leptomeningeal lymphatic endothelial cells (LLECs) have been less studied functionally, which were firstly harvested and cultured *in vitro* by our group previously and are probably related to the clearance of extravasated erythrocytes post-SAH for they closely surround subarachnoid space.

**Methods:** We established a SAH animal model, employed primary LLECs *in vitro*, mimicked the conditions of the SAH *in vitro*, performed RNA sequencing, and transfected LLECs with adenovirus and adeno-associated virus both *in vivo* and *in vitro* to reveal the molecular mechanisms of efferocytosis of erythrocytes by LLECs and its neuroprotection post-SAH.

**Results:** Firstly, we demonstrated the eryptosis-initiated degradation of extravasated erythrocytes *in vitro*. Furthermore, we found LLECs preferentially adhered and engulfed apoptotic erythrocytes *in vivo* and* in vitro* while sparing from intact erythrocytes, suggesting their novel capacity in the efferocytosis of erythrocytes. Additionally, the efferocytosis of erythrocytes by LLECs plays a role on neuroprotection via improving neurological functions, maintaining neurostructural integrity, and alleviating neuropathological consequences post-SAH. During efferocytosis, phosphatidylserine (PS) and phosphatidylserine receptor (PSR) mediated the recognition of apoptotic erythrocytes by LLECs. We also confirmed that NHL repeat-containing 2 (NHLRC2) positively regulated the efferocytosis of erythrocytes by LLECs to serve as a central regulator in it mediated neuroprotection post-SAH.

**Conclusions:** This study elucidated the efferocytosis of erythrocytes by LLECs and subsequently neuroprotection post-SAH. These findings highlight a prompt, efficient, and regulable pathway for the autologous clearance of extravasated erythrocytes that performs as a sentinel against brain injury post-SAH.

## Introduction

Subarachnoid hemorrhage (SAH) is characterized by sudden bleeding into the subarachnoid space (SAS), typically arising from a ruptured cerebral aneurysm 1. Although the overall incidence of SAH worldwide is approximately 8 per 100,000 person-years 2, SAH is considered a medical emergency due to its associated complications and life-threatening nature 1. There is an urgent need to elucidate the pathophysiological mechanisms of SAH to improve prognosis 3. Early brain injury occurs within the first 72 hours post-SAH and has a long-lasting impact on mortality 4. Extravasated erythrocytes disperse throughout the SAS after SAH 5. Erythrocytes and degradative products, which are released into the cerebrospinal fluid (CSF), are the primary instigators of brain injury and contribute to an unfavorable prognosis 6, 7. Hence, mitigating erythrocyte-derived brain injury, as a pivotal modulator to improve prognosis is attracting increasing attention. Currently, the clearance of erythrocytes and degradative products is mainly thought to involve phagocytosis and drainage. Resident parenchymal microglia mediate the phagocytosis of erythrocytes and degradative products and do not appear to be directly involved in the clearance of erythrocytes within the SAS 5, 8. Infiltrating macrophages have been proven to predominantly facilitate the clearance of erythrocytes at 2 days post-SAH 5. The glymphatic system and meningeal lymphatic vessels drain erythrocytes and degradative products to the cervical lymph nodes (CLNs), accompanied by persistent dysfunction and related neuropathological damage 9-16. Consequently, the pathways for the autologous clearance of extravasated erythrocytes post-SAH are limited by their indirect, unpunctual, and inefficient nature. Exploring a novel pathway for the autologous clearance of extravasated erythrocytes that prevents degradation represents the optimal approach against brain injury and serves as an ideal alternative to improve prognosis.

Erythrocytes can undergo apoptosis in an abnormal state or at an abnormal site without the release of intracellular substances through a process termed eryptosis 17. Efferocytosis is the process by which apoptotic cells are recognized and engulfed by efferocytes 18, 19. Previous research has indicated that apoptotic erythrocytes are cleared via efferocyte-mediated efferocytosis, preventing the release of degradative products and thus contributing to recovery following intracerebral hemorrhage 20, 21. The leptomeninges comprise the pia mater and the arachnoid layer 22. Leptomeningeal lymphatic endothelial cells (LLECs) represent a unique intracranial cellular subpopulation and form a meshwork of individual cells 23. Furthermore, LLECs have been shown to clear macromolecules from the CSF, an ability reminiscent of that of scavenger cells 23. LLECs have been less studied functionally, which were firstly harvested and cultured *in vitro* by our group previously and are probably related to the clearance of extravasated erythrocytes post-SAH for they closely surround SAS 24. However, the mechanisms underlying recognition and engulfment during efferocytosis, through which LLECs clear extravasated erythrocytes, post-SAH remain largely unknown.

Accordingly, we specifically investigated the efferocytosis of erythrocytes by LLECs, elucidating its contributions post-SAH. First, we confirmed the anatomical distribution of LLECs and verified their capacity to phagocytose substances in the CSF. We also found the eryptosis-initiated degradation of extravasated erythrocytes in artificial cerebrospinal fluid (ACSF). Our data revealed the mechanisms of recognition and engulfment during the efferocytosis of erythrocytes by LLECs. Finally, we discovered the neuroprotection of LLECs-mediated efferocytosis post-SAH. Overall, this study elucidated the efferocytosis of erythrocytes by LLECs and subsequently neuroprotection post-SAH.

## Methods

### Experimental design

**Figure 1:**
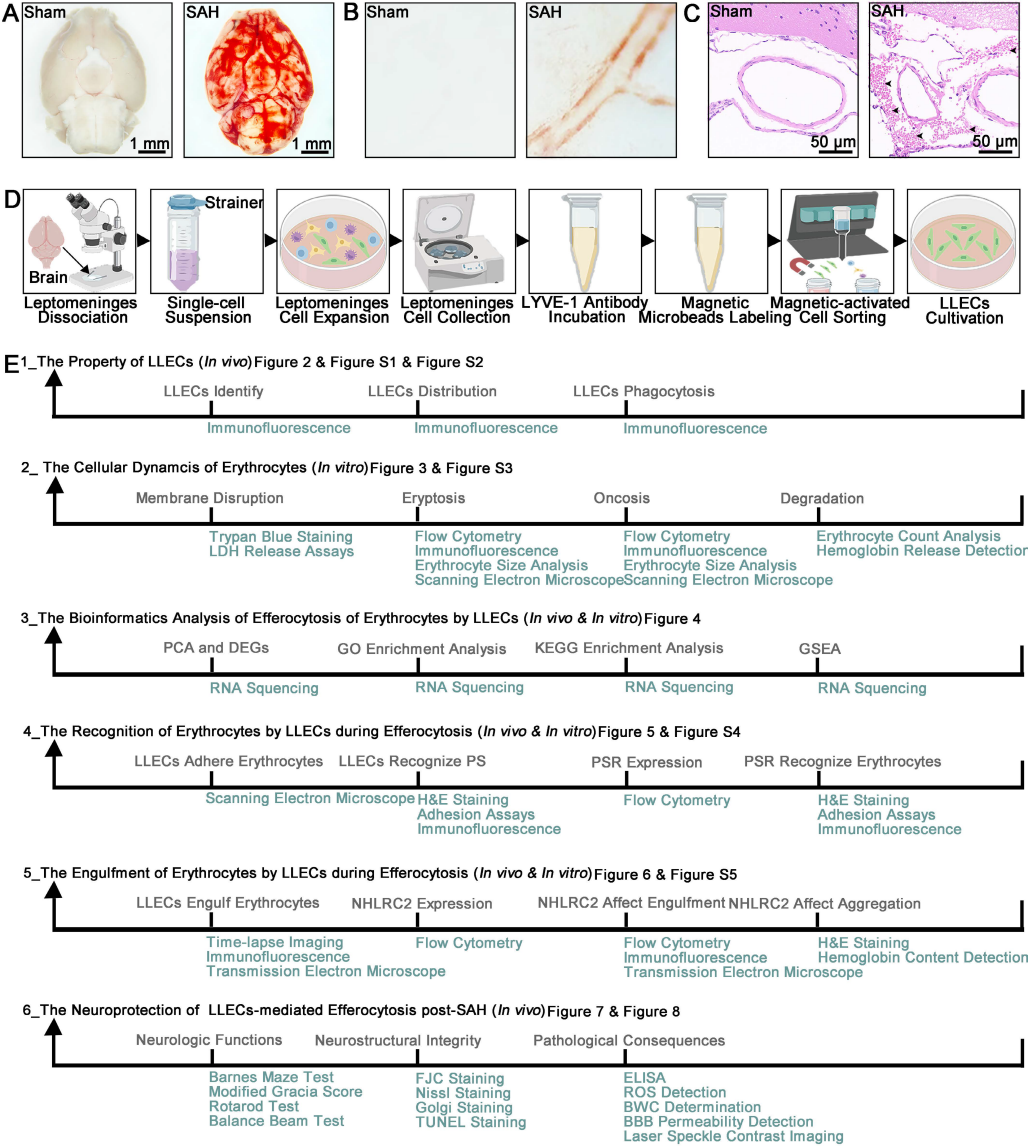
** SAH animal model, multistep primary LLECs culture, and experimental framework. (A)** Observation of ventral brain tissue in the sham and SAH groups, with blood deposition specifically in the SAH group (scale bars: 1 mm).** (B)** Observation of the brain surface vasculature in the sham and SAH groups revealed blood accumulation around the vasculature in the SAH group. **(C)** Representative H&E staining images of the sham and SAH groups demonstrated that blood accumulated in the SAS (erythrocytes are indicated by black arrows; scale bars: 50 μm). **(D)** Schematic of the multiple procedures for harvesting and culturing primary LLECs. **(E)** Schematic overview of the study topic and corresponding experimental approaches.

Tissue or cell samples were obtained from mice. The sample size was determined based on prior studies of this type conducted by others, as detailed in each figure legend. To ensure reliable replication, experimental methods and analysis were designed to include multiple technical and biological replicates. Following the establishment of an SAH mice model and a procedure for culturing primary LLECs. Light microscopy images showed that blood deposition in the ventral brain tissue and around the vasculature specifically in the SAH group (Figure 1A-B). Representative hematoxylin and eosin (H&E) staining images of SAH groups demonstrated that blood accumulated in the SAS (Figure 1C). The multiple procedures were carried out for harvesting and culturing primary LLECs (Figure 1D). We conducted a total of six experiments to investigate the efferocytosis of erythrocytes by LLECs and its contributions post-SAH (Figure 1E).

(1) Part 1 of the experiment. Immunofluorescence staining was employed to identify various LLEC markers, distinct from macrophage, and confirm the anatomical distribution of LLECs in relation to the arachnoid layer, astrocytes, vascular endothelial cells, pia mater, and perivascular spaces. Immunofluorescence staining was also performed to verify the phagocytosis of LLECs after injecting dextran-3 kDa, β-amyloid, or microspheres-6 μm into the CSF and establishing an SAH model. Immunofluorescence staining was also performed to verify the distribution of dextran-3 kDa and erythrocyte in relation to the vascular endothelial cells, pia mater, and perivascular spaces. The mice were randomly divided into the following groups: the control group, dextran-3 kDa 1-hour group, dextran-3 kDa 12-hour group, and dextran-3 kDa 24-hour, β-amyloid group, microspheres-6 μm group, SAH 1-hour group, 6-hour group, SAH 9-hour group, SAH 12-hour group and SAH 24-hour group.

(2) Part 2 of the experiment. Erythrocyte counts analysis, hemoglobin release detection, trypan blue staining, lactate dehydrogenase (LDH) release assays, scanning electron microscope, erythrocyte size analysis, immunofluorescence staining, and flow cytometry were utilized to investigate the cellular dynamics of erythrocytes in ACSF. Erythrocyte were randomly divided into the following groups: the 0-hour group, 6-hour group, 12-hour group, 24-hour group, 2-day group, and 3-day group.

(3) Part 3 of the experiment. Differentially expressed genes (DEGs) analysis, principal component analysis (PCA), gene ontology (GO) enrichment analysis, kyoto encyclopedia of genes and genomes (KEGG) enrichment analysis, and gene set enrichment analysis (GSEA) were utilized to investigate RNA sequencing data in LLECs. The LLECs were randomly divided into the following groups: the control group, red blood cell (RBC) group, RBC + NHLRC2 (KD) group, the sham group, and SAH group. As a comparison for the RBC group, the control group received no erythrocyte stimulation. The RBC + NHLRC2 (KD) group was treated with shRNA-adenovirus (AV) for NHLRC2 knockdown. The sham group was injected with the same volume of normal saline as the SAH group.

(4) Part 4 of the experiment. Scanning electron microscope, H&E staining, adhesion assays, immunofluorescence staining, reverse transcription-quantitative polymerase chain reaction (RT-qPCR), and flow cytometry were used to determine the mechanism by which LLECs recognize apoptotic erythrocytes during efferocytosis. The LLECs were randomly divided into the following groups: the control group, 3-hour group, 6-hour group, RBC group, RBC + vehicle group, RBC + IgG group, RBC + PS antibody group, RBC + PS liposome group, RBC + PSR (OE_NC) group, RBC + PSR (KD_NC) group, RBC + PSR (KD) group, and RBC + PSR (OE) group. As a comparison for the RBC group, the control group received no erythrocyte stimulation. The RBC + PS antibody and RBC + PS liposome groups were treated with PS antibody and PS liposomes to block PS or encapsulate erythrocytes with PS, respectively. The RBC + vehicle group was treated with the same volume of phosphate-buffered saline (PBS) for comparison to the RBC + PS liposome group. The RBC + IgG group was treated with an equal volume of IgG for comparison with the RBC + PS antibody group. The RBC + PSR (KD) and RBC + PSR (OE) groups were treated with shRNA-adenovirus (AV) and recombinant AV for PSR knockdown and overexpression, respectively. The RBC + PSR (OE_NC) group was treated with an equal volume of unloaded AV for comparison with the RBC + PSR (OE) group. The RBC + PSR (KD_NC) group was treated with an equal volume of scrambled shRNA-AV for comparison with the RBC + PSR (KD) group. The mice were randomly divided into the following groups: the sham group, SAH group, SAH + vehicle group, SAH + IgG group, SAH+PS antibody group, and SAH + PS liposomes group. The sham group was injected with the same volume of normal saline as the SAH group. The SAH + PS antibody and SAH + PS liposome groups were treated with PS antibody and PS liposomes to block PS and encapsulate erythrocytes with PS, respectively. The SAH + vehicle group was treated with the same volume of PBS for comparison with the SAH + PS liposomes group. The SAH + IgG group was treated with the same volume of IgG for comparison with the SAH + PS antibody group.

(5) Part 5 of the experiment. Transmission electron microscope (TEM), time-lapse imaging, immunofluorescence staining, RT-qPCR, flow cytometry, H&E staining, and hemoglobin content detection were performed to explore the efferocytosis of erythrocyte by LLECs. The erythrocytes were randomly divided into the following groups: the control group, vehicle group, and ionomycin group. The control group received no treatment for comparison with the vehicle group. The ionomycin group was treated with ionomycin to induce eryptosis. The vehicle group was treated with the same volume of Ringer's solution for comparison with the ionomycin group. The LLECs were randomly divided into the following groups: the control group, 6-hour group, 9-hour group, 12-hour group, RBC group, RBC + vehicle group, RBC + ionomycin group, RBC + NHLRC2 (OE_NC) group, RBC + NHLRC2 (KD_NC) group, RBC + NHLRC2 (KD) group, and RBC + NHLRC2 (OE) group. As a comparison for the RBC group, the control group received no erythrocyte stimulation. The RBC + ionomycin group was treated with ionomycin to induce eryptosis. For comparison with the RBC + ionomycin group, the RBC + vehicle group was treated with the same volume of Ringer's solution. The RBC + NHLRC2(KD) and RBC + NHLRC2(OE) groups were treated with shRNA-AV and recombinant-AV for NHLRC2 knockdown and overexpression, respectively. The RBC + NHLRC2(OE_NC) group was treated with the same volume of unloaded AV for comparison to the RBC + NHLRC2(OE) group. For comparison with the RBC + NHLRC2 (KD) group, the RBC + NHLRC2 (KD_NC) group was treated with the same volume of scrambled shRNA-AV. The mice were randomly divided into the following groups: the sham group, SAH group, SAH + NHLRC2 (OE_NC) group, SAH + NHLRC2 (KD_NC) group, SAH + NHLRC2 (KD) group, and SAH + NHLRC2 (OE) group. The sham group was injected with the same volume of normal saline as the SAH group. The SAH + NHLRC2(KD) and SAH + NHLRC2(OE) groups were injected with shRNA- adeno-associated virus (AAV) and recombinant AAV for NHLRC2 knockdown and overexpression, respectively. The SAH + NHLRC2(OE_NC) group was injected with the same volume of unloaded AAV for comparison to the SAH + NHLRC2(OE) group. The SAH + NHLRC2(KD_NC) group was injected with the same volume of scrambled shRNA-AAV for comparison with the SAH + NHLRC2(KD) group.

(6) Part 6 of the experiment. The modified Garcia score test, beam balance test, Barnes maze test, rotarod test, Nissl staining, terminal deoxynucleotidyl transferase-mediated dUTP nick-end labeling (TUNEL) staining, Golgi staining, fluoro-Jade C (FJC) staining, laser speckle contrast imaging, reactive oxygen species (ROS) detection, brain water content determination, blood-brain barrier integrity evaluation, and enzyme-linked immunosorbent assay (ELISA) were conducted to evaluate the contributions of NHLRC2 in LLECs post-SAH. The mice were randomly divided into the following groups: the sham group, SAH group, SAH + NHLRC2 (OE_NC) group, SAH + NHLRC2 (KD_NC) group, SAH + NHLRC2 (KD) group, and SAH + NHLRC2 (OE) group. The sham group was injected with the same volume of normal saline as the SAH group. The SAH + NHLRC2(KD) and SAH + NHLRC2(OE) groups were injected with shRNA-AAV and recombinant AAV for NHLRC2 knockdown and overexpression, respectively. The SAH + NHLRC2(OE_NC) group was injected with the same volume of unloaded AAV for comparison to the SAH + NHLRC2(OE) group. The SAH + NHLRC2(KD_NC) group was injected with the same volume of scrambled shRNA-AAV for comparison with the SAH + NHLRC2(KD) group.

Detailed descriptions of our study design, methodologies, and statistical approaches are presented in the figures, figure legends, and supplemental data file. Data collection was terminated when mice completed the predetermined experimental plan in compliance with the Guide for the Care and Use of Laboratory Animals of the National Institutes of Health. ARRIVE guidelines were followed throughout the study design. All animal procedures were approved by the Animal Experiment Ethics Committee of Kunming Medical University (kmmu20220945).

### Mice

C57BL/6J male mice (10-12 weeks old) weighing 20-25 g were obtained from Kunming Medical University in Kunming, China. All mice were housed and maintained under specific pathogen-free conditions on a regular 12-hour light/dark cycle and in a temperature-controlled environment (26 ± 2 °C). The animals received food and water ad libitum and were allowed to adapt to the environment for 1 week before the experiment.

### SAH animal model

The SAH animal model was generated as previously described 25. Briefly, the mice were anesthetized with 2% isoflurane in 100% O_2_, and anesthesia was maintained with 1% isoflurane. The head of each mouse was fixed in a stereotactic apparatus. The skin overlying the anterior skull was opened with a midline incision. A burr hole was drilled 4.5 mm anterior to the bregma using a 0.9 mm drill until the dura was penetrated. A 27-gauge needle was advanced ventrally at a 40° angle through the burr hole. Then, 50 μL of arterial blood was injected into the prechiasmatic cistern over 10 seconds. The needle was left in place for 3 minutes and then slowly removed to avoid backflow. The burr hole was immediately sealed with bone wax, and the incision was sutured. The mice were observed for recovery on a heating pad until they were fully awake before being returned to their cages. Sham animals underwent the same procedures without blood injection.

### Primary LLECs culture

The primary LLECs were cultured as we previously reported in detail 24. Leptomeninges were dissected from adult mice using fine-point tweezers under a microscope. The leptomeningeal tissue was cut into fragments, digested with an enzyme mixture, and filtered with a 70 μm strainer to remove any clumps to obtain a single-cell suspension. The cells were incubated for 24 hours, and the medium was removed to eliminate unattached cells for subsequent cell expansion. 0.25% trypsin was added to detach the adherent cells, and the cells were centrifuged at 300 × g for 5 minutes for collection. The cells were incubated with 10 μL of LYVE-1 antibody (12-0443-82, eBioscience) for 30 minutes in the dark at 4 °C. Then, 20 μL of magnetic microbeads (130-048-801, Miltenyi Biotec) were added and incubated for 30 minutes in the dark at 4 °C. LYVE-1-positive LLECs were collected and resuspended in medium according to the manufacturer's instructions for magnetic-activated cell sorting. LLECs were utilized for subsequent experiments after 2-3 passages.

### Intracerebroventricular injection

Intracerebroventricular injection was performed as previously described 26. Briefly, the mice were placed in a stereotactic apparatus after being anesthetized with 2% isoflurane in 100% O_2_ and maintained with 1% isoflurane. Then, a midline incision was made in the skin. A Hamilton syringe was inserted into the ventricles through a small cranial burr hole, 0.3 mm posterior, 1.0 mm right lateral, and 3 mm deep relative to the bregma. The desired volume of each solution was injected into the intracerebroventricular compartment. The syringe was left *in situ* for an additional 5 minutes after the injection and then slowly removed to prevent CSF leakage and backflow. The burr hole was immediately sealed with bone wax, and the incision was sutured. The mice were observed for recovery on a heating pad until fully awake before being returned to their cages.

### AAV delivery and AV transfection

(1) For the specific overexpression or knockdown of NHLRC2 in LLECs *in vivo*, adeno-associated virus serotype 9 (AAV9) with the LLEC-specific promoter of LYVE-1 (ENSMUST00000033050.5 range=chr7:110462447-110464446) was constructed. A total of 5 μL (1x10^12^ vg/ml) of recombinant AAV (NM_025811.3) or shRNA-AAV (targeting sequence:5'-CCGGAAGGCTTAGAATGGCTGAACA-3') was injected into the lateral ventricle at a rate of 0.2 μL/min. Unloaded AAV or scrambled shRNA-AAV was used as the negative control for the overexpression or knockdown group, respectively. The SAH animal model was established 3 weeks after AAV injection. (2) For the overexpression or knockdown of NHLRC2 *in vitro*, LLECs were transfected with recombinant AV (NM_025811.3) or shRNA-AV (targeting sequence: 5'-CCGGAAGGCTTAGAATGGCTGAACA-3'), respectively. For the overexpression or knockdown of PSR *in vitro*, LLECs were transfected with recombinant AV (NM_033398.3) or shRNA-AV (targeting sequence: 5'-GTTATCAAGGAAGTGGTATAG-3'), respectively. Unloaded AV or scrambled shRNA-AV was used as the negative control group for the overexpression or knockdown group, respectively. The LLECs were transfected with AV (1x10^10^ PFU/ml; multiplicity of infection [MOI] = 30) for 48 hours before further experiments. All AAVs and AVs were purchased from Hanbio Biotechnology (Shanghai, China).

### Drug administration

(1) Apoptotic erythrocytes were induced as described previously 27. Erythrocytes were treated with 1 μM ionomycin (I3909, Sigma‒Aldrich) in Ringer's solution (BR0052G, Thermo Fisher Scientific) for 3 hours at 37 °C. Apoptotic erythrocytes were detected by labeling with an Annexin V conjugate. Ringer's solution was used as the negative control for ionomycin. (2) Erythrocytes were encapsulated in 1 mM PS liposomes (CPS-514, Sigma‒Aldrich) dissolved in PBS for 3 hours at 37 °C before subsequent downstream processing, as described in a previous study 28. The same volume of PBS was used as the negative control treatment for PS liposomes. (3) A 20 μg/mL concentration of PS antibody (05-719, Sigma‒Aldrich) was used for 2 hours to block PS in erythrocytes, as previously reported 29. IgG (16-4714-85, Life Technologies) was used as the negative control treatment for the PS antibody.

### RNA sequencing

Magnetic-activated cell sorting was performed to obtain LYVE-1-positive LLECs as we previously reported^24^. Total RNA was extracted from LLECs using the TRIzol reagent (R0016, Beyotime). Strand-specific mRNA-seq libraries for the Illumina NovaSeq-PE150 platform were generated and sequenced. PCA was performed with the R package. DESeq2 was used to normalize the counts and perform differential expression analysis. DEGs were defined as genes with |log2(fold change) | > 2 and with a Benjamini-Hochberg adjusted p-value ≤ 0.05. DEGs identified were submitted to the GO database for GO enrichment analysis and the KEGG database for KEGG enrichment analysis. GSEA was performed using GSEA software to identify whether a set of genes was enriched in specific GO terms or KEGG pathways. The RNA sequencing data generated in this study has been deposited in the Genome Sequence Archive (GSA) database under the accession number CRA021707.

### Incubation of erythrocytes in ACSF

Erythrocytes were extracted from whole blood and collected by centrifugation at 300 × g for 5 minutes. Erythrocytes were incubated in ACSF (CZ0540, Leagene) at 37 °C to mimic the conditions of the SAH *in vitro* for various durations for subsequent experiments.

### Adhesion assays

Adhesion between erythrocytes and LLECs was evaluated as previously described, with some modifications 30. Briefly, LLECs were co-incubated with erythrocytes for 6 hours, and then the nonadherent erythrocytes were removed by washing the monolayers three times with PBS. The percentage of erythrocyte adhesion ratio was calculated using the following formula: [(total number of erythrocytes - number of nonadherent erythrocytes) / total number of erythrocytes] × 100%. The negative control group was not treated with erythrocytes.

### Phagocytosis assays

(1) To evaluate LLECs phagocytosis *in vivo*, mice were anesthetized with 2% isoflurane in 100% O_2_ and maintained at 1% isoflurane. A stereotactic apparatus was used to stabilize the head, and a midline incision was made in the skin. The muscle layers were retracted to expose the cisterna magna. A Hamilton syringe was inserted into the cisterna magna to inject dextran-3 kDa (D3307, Invitrogen), β-amyloid (ab120957, Abcam), or microspheres-6 μm (F14806, Invitrogen). (2) The engulfment of erythrocytes by LLECs *in vitro* was observed as previously described, with some modifications 31. Briefly, erythrocytes were resuspended in medium supplemented with LLECs for 12 hours. The LLECs were thoroughly washed with PBS three times to collect free erythrocytes, and LLECs were obtained after 0.25% trypsin digestion. Images were acquired with a fluorescence confocal microscope (N-SIM E, Nikon, Japan) and a TEM (HT7800, Hitachi, Japan). The engulfment index was calculated as the percentage of engulfed LLECs out of the total LLECs by flow cytometry. The control group was not treated with erythrocytes. (3) To record a dynamic video of phagocytosis *in vitro*, images were collected for 3.5 hours by time-lapse microscope after the LLECs were co-incubated with erythrocytes for 6 hours.

### Immunofluorescence staining

Immunofluorescence staining of brain cryostat slices and cell slices was performed as described previously 25. Briefly, the slices were fixed in 4% paraformaldehyde. The slices were blocked with serum for 2 hours and then incubated at 4 °C overnight with the following primary antibodies: LYVE-1 (1:100, ab14917, Abcam), PROX1 (1:100, sc-81983, Santa), VEGFR-3 (1:100, sc-514825, Santa), PDPN (1:100, sc-53533, Santa), E-cadherin (1:100, ab231303, Abcam), PECAM-1 (1:100, ab24590, Abcam), AQP-4 (1:100, ab259318, Abcam), F4/80 (1:100, sc-377009, Santa), Plectin (1:100, sc-33649, Santa), TER-119 (1:100, ab93587, Abcam; 1:100, ab93582, Abcam), Annexin V (1:100, ab14085, Abcam), and PORIMIN (1:100, sc-377295, Santa). On the second day, the slices were incubated with fluorescence-conjugated secondary antibodies for 1 hour at room temperature. Finally, the slices were counterstained with 4,6-diamidino-2-phenylindole (DAPI) (P0131, Beyotime) at room temperature for 3 minutes. The images were visualized under a fluorescence confocal microscope (N-SIM E, Nikon).

### Erythrocyte counts and size analysis

Erythrocyte counts and sizes were determined using an automatic cell counter (AMQAX2000, Thermo Fisher Scientific) according to the manufacturer's instructions.

### Hemoglobin release and content detection

Hemoglobin release and content were measured according to the manufacturer's instructions, as previously described 32, 33. (1) Erythrocytes in ACSF were centrifuged at 300 × g for 5 minutes to harvest the supernatant. Hemoglobin release into the supernatant was measured and converted into the percentage of hemolysis. The absorption of the supernatant from erythrocytes lysed in distilled water was defined as 100% hemolysis. (2) CLNs and brain tissue were isolated 12 hours post-SAH and processed for homogenization. Hemoglobin content in the supernatant and homogenate were determined by using an established standard curve. The optical density (OD) values of hemoglobin in the supernatant and homogenate were measured at 540 nm after reacting with Drabkin's reagent (D5941, Sigma‒Aldrich) for 15 minutes.

### Trypan blue staining

Trypan blue staining was used to detect erythrocyte membrane disruption based on a previous study 34. Briefly, erythrocytes in ACSF were collected after centrifugation at 300 × g for 5 minutes. A 0.4% trypan blue solution (T10282, Invitrogen) was incubated with the erythrocytes at 37 °C for 30 minutes. The percentage of trypan blue-positive erythrocyte was calculated using an automatic cell counter (AMQAX2000, Thermo Fisher Scientific).

### LDH release assays

Erythrocyte membrane disruption was also monitored by measuring LDH release into the ACSF using an LDH assay kit (C0016, Beyotime) according to the manufacturer's protocol, as previously described 35. Briefly, erythrocytes in ACSF were centrifuged at 300 × g for 5 minutes to harvest the supernatant. The absorption of the supernatant from erythrocyte lysed in distilled water was defined as 100% release. The supernatant was reacted with the LDH detection working fluid for 1 hour in the dark, and the OD values of LDH in the supernatant were measured at 490 nm.

### Scanning electron microscope

Erythrocytes in ACSF and LLECs on cell slices were collected and fixed in 2.5% glutaraldehyde for 2 hours at room temperature. The cells were washed with PBS three times and transferred to 1% osmium tetroxide for 2 hours at room temperature in the dark. The cells were dehydrated for 15 minutes in sequential concentrations of 50%, 70%, 80%, 90%, and 100% ethanol and finally in isoamyl acetate. The cells were dried using a critical point dryer (K850, Quorum, UK), attached to metallic stubs using carbon stickers and sputter-coated with gold for 30 seconds before visualizing the morphology of erythrocytes and LLECs with a scanning electron microscope (SU8100, Hitachi, Japan).

### Flow cytometry

Single-cell suspensions of erythrocytes or LLECs were obtained and subjected to staining with fluorescently labeled antibodies against the following proteins in staining buffer for 30 minutes: TER-119 (1:100, ab93582, Abcam), Annexin V (1:100, ab14085, Abcam), PORIMIN (1:100, sc-377295, Santa), PSR (1:100, sc-28349, Santa), NHLRC2 (1:100, orb185631, Biorbyt), and LYVE-1 (1:100, 12-0443-82, eBioscience). The data were acquired on a flow cytometer (BD Biosciences, USA). The percentage of positive markers and the mean fluorescence intensity (MFI) were calculated using FlowJo software version 7.6.1. Single stain positive control and fluorescence minus one (FMO) negative control were used to help determine the compensation matrix and properly set the gates.

### RT-qPCR

Magnetic-activated cell sorting was performed to obtain LYVE-1-positive LLECs as we previously reported^24^. Total RNA was isolated using Trizol (R0016, Beyotime) according to the manufacturer's protocol. The RNA was reverse transcribed using a first-strand cDNA synthesis kit (Takara, Dalian, China). RT-qPCR was conducted using the following specific primers: PSR-forward 5'-CACCAACTTCCCTGTTGTGTG-3', PSR-reverse 5'-CCTGGAGGTCAACTGCGTC-3'; NHLRC2-forward 5'-GGAAGGCTTAGAATGGCTGAAC-3', NHLRC2-reverse 5'-CAGTTTATGCAGCAGTAGGTGA-3'; GAPDH-forward 5'-AGATCCCTCCAAAATCAAGTGG-3', GAPDH-reverse 5'-GGCAGAGATGATGACCCTTTT-3'. RT-qPCR was performed using FastStart Universal SYBR Green Master (Roche, Mannheim, Germany). The expression levels of relative mRNA of genes were determined by the 2^-ΔΔCT method.

### H&E staining

Brain cryostat slices, CLNs cryostat slices, and cell slices were used for H&E staining according to the routine protocol 36. Briefly, slices were stained with hematoxylin for 5 minutes and eosin for 10 seconds and visualized by a microscope. The number of erythrocytes in the SAS was calculated by ImageJ software (NIH, USA).

### TEM

LLECs were harvested after 0.25% trypsin digestion and then fixed in 2.5% glutaraldehyde. The samples were washed with PBS three times and then transferred to 1% osmium tetroxide for 2 hours at room temperature in the dark. The sample was dehydrated for 15 minutes in sequential concentrations of 50%, 70%, 80%, 90%, and 100% ethanol and finally in acetone. Subsequently, the sample was infiltrated with resin, embedded in acetone and EMBed 812 overnight at 37 °C, mounted on grids, and stained with uranyl acetate and lead citrate under CO₂-free conditions. The morphology of the LLECs was observed via TEM (HT7800, Hitachi, Japan).

### Modified Garcia score test

Short-term neurological function was evaluated 24 hours post-SAH utilizing a previous modified Garcia score test 37. The evaluation scores ranged from 3 to 18 and consisted of 6 aspects: spontaneous activity (0-3 points), symmetry in the movement of all four limbs (0-3 points), forelimb outstretching (0-3 points), climbing ability (1-3 points), body proprioception (1-3 points), and the response to vibrissae stimulation (1-3 points). Higher scores indicated less severe neurological deficits. The assessments were all performed at the same time of day.

### Beam balance test

Short-term neurological function was assessed 24 hours post-SAH via the balance beam test 38. The scores ranged from 0 to 6, and the scoring standards were as follows: maintaining a stable balance (0 points); grasping one end of the balance beam (1 point); clinging to the balance beam, with one side of the limb slipping (2 points); holding the balance beam, with both sides of the body rotating around the wood or falling after more than 60 seconds (3 points); trying to maintain balance but slipping after between 40 and 60 seconds (4 points); trying to maintain balance but slipping after between 20 and 40 seconds (5 points); and unable to maintain balance and slipping within 20 seconds (6 points). A higher score represented more severe neurobehavioral deficits. The assessments were all performed at the same time of day.

### Nissl staining

Nissl staining was performed to evaluate the functional state of neurons 24 hours post-SAH in the hippocampus, as previously described 39. Briefly, brain cryostat slices were dehydrated in ethanol and then stained with cresyl violet (C0117, Beyotime) for 5 minutes. Neurons were observed under a light microscope. Compared with those of normal neurons, the cell bodies of damaged neurons appeared to be shrunken and to have condensed staining. The percentage of normal neurons index was calculated using the following formula to assess the neuronal state: [(total number of neurons - number of damaged neurons) / total number of neurons] × 100%.

### TUNEL staining

The apoptosis of neural cells was monitored using a TUNEL kit (11684795910, Roche) 24 hours post-SAH based on a previous method according to the manufacturer's protocol 40. The brain cryostat slices were incubated with the TUNEL reaction mixture for 1 hour at 37 °C and then counterstained with DAPI. The samples were subsequently observed under a fluorescence microscope. The severity of brain injury was evaluated using the apoptosis index, which is defined by the percentage of TUNEL-positive cells, according to ImageJ software (NIH, USA). The percentage of TUNEL-positive cells was calculated as [(number of TUNEL-positive cells/total number of cells)] × 100%.

### Barnes maze test

Long-term neurological function was assessed 14 days post-SAH using the Barnes maze test, as previously described 41. A dark box was placed under one of the holes as an escape box. The mice were allowed to remain in the box for 30 seconds once they entered the escape box. The results were recorded as the escape latency time (time needed to find the escape box). A longer time indicated worse neurological function. The assessments were all performed at the same time of day.

### Rotarod test

Long-term neurological function was assessed 14 days post-SAH using the rotarod test 42. Briefly, the initial speed of the rotating rod was 0 rpm, the acceleration was 1 rpm/s, and the maximum speed was 60 rpm. The results were recorded as the duration of time spent on the rotarod and were used for statistical analysis. A shorter time corresponded to a worse neurobehavioral effect. The assessments were all performed at the same time of day.

### Golgi staining

Golgi staining was performed to evaluate the synaptic function of neurons at 14 days post-SAH with an FD Rapid GolgiStain Kit (PK401, FD Neuro Technologies) according to the manufacturer's protocol, as previously reported 43. The dendritic spine density was quantified as the number of spines per 10 μm using ImageJ software (NIH, USA).

### FJC staining

The temporal lobe cortex was evaluated with an FJC staining kit (TR-100-FJT, Biosensis) to identify degenerating neurons at 14 days post-SAH, according to a previous method 44. The brain cryostat slices were treated with xylene, followed by gradient ethanol. The prepared glacial acetic acid was combined with FJC working liquid, and the slices were incubated overnight at 4 °C in the dark. Then, the nuclei were stained with DAPI for 3 minutes. Images were captured with a fluorescence microscope. The FJC-positive cells were counted manually using ImageJ software (NIH, USA). The percentage of FJC-positive cells was calculated as [(number of FJC-positive cells/total number of cells)] × 100%.

### Laser speckle contrast imaging

Laser speckle contrast imaging was performed utilizing laser speckle flowmetry to instantaneously visualize brain blood perfusion in the circulation 24 hours after SAH, as described in a previous study 9. All measurements were performed under anesthesia with 2% isoflurane in 100% O_2_ while maintaining the body temperature at 37 °C on a constant heating pad. In brief, the head was immobilized in a stereotactic apparatus. A midline incision was made to expose the calvaria. RFLSI laser speckle (RWD Life Science, China) was used to calculate the mean blood flow index in real time and in the vascular area within the regions of interest. The corresponding images were analyzed using the software installed in the laser speckle imaging system.

### ROS detection

Brain tissue ROS levels were investigated 24 hours post-SAH using DHE staining according to the manufacturer's instructions following a previously described method 45. In brief, fresh brain slices were incubated with DHE solution (D7008, Sigma‒Aldrich) for 30 minutes at 37 °C in the dark, followed by staining with DAPI for 5 minutes. Images were captured with a fluorescence microscope. The fluorescence density was quantified using ImageJ software (NIH, USA).

### Brain water content determination

The brain water content was measured using the wet‒dry method according to previously reported procedures 46. Briefly, the whole brain was harvested 24 hours post-SAH and weighed to obtain the wet weight values. The brain was subsequently placed in an oven for 72 hours at 80 °C to obtain the dry weight. The percentage of brain water content was calculated using the following formula: [(wet weight - dry weight) / wet weight] × 100%.

### Blood-brain barrier integrity detection

Blood-brain barrier integrity was assessed 24 hours post-SAH, as described in a previous study 47. Evans blue dye (2%, 4 ml/kg, E2129, Sigma‒Aldrich) was injected intraperitoneally, and the solution was allowed to circulate for 3 hours before the mouse was decapitated. Mice were anesthetized and perfused with a normal saline solution to remove the circulating dye. The brain tissue was homogenized in 50% trichloroacetic acid and then incubated overnight at 4 °C. The OD values of the supernatants were measured at 620 nm.

### ELISA

Commercial ELISA kits (88-7324, 88-7013, 88-7064, and 88-7105; Thermo Fisher Scientific) were used to evaluate the concentrations of tumor necrosis factor-alpha (TNF-α), interleukin (IL)-1β, IL-6, and IL-10 in the serum 24 hours post-SAH. All procedures were performed according to the manufacturer's instructions, as previously described 25. The OD values were measured at 450 nm.

### Statistical analysis

The allocation of animals to experimental groups was randomized, as were the *in vitro* studies. All analyses were performed blinded by two investigators. All statistical analyses were performed using GraphPad Prism software version 8. The data are expressed as the mean ± standard error of the mean (SEM). The Kolmogorov‒Smirnov test was used to assess the normality of the experimental data. Two groups were compared with an unpaired two tailed Student's t test or the Mann‒Whitney U test. Multiple groups were compared using one-way analysis of variance (ANOVA) with Dunnett's test. A p value less than 0.05 was considered to indicate significance.

## Results

### LLECs surround the SAS and phagocytize dextran-3 kDa, β-amyloid, microspheres-6 μm, and erythrocytes

**Figure 2:**
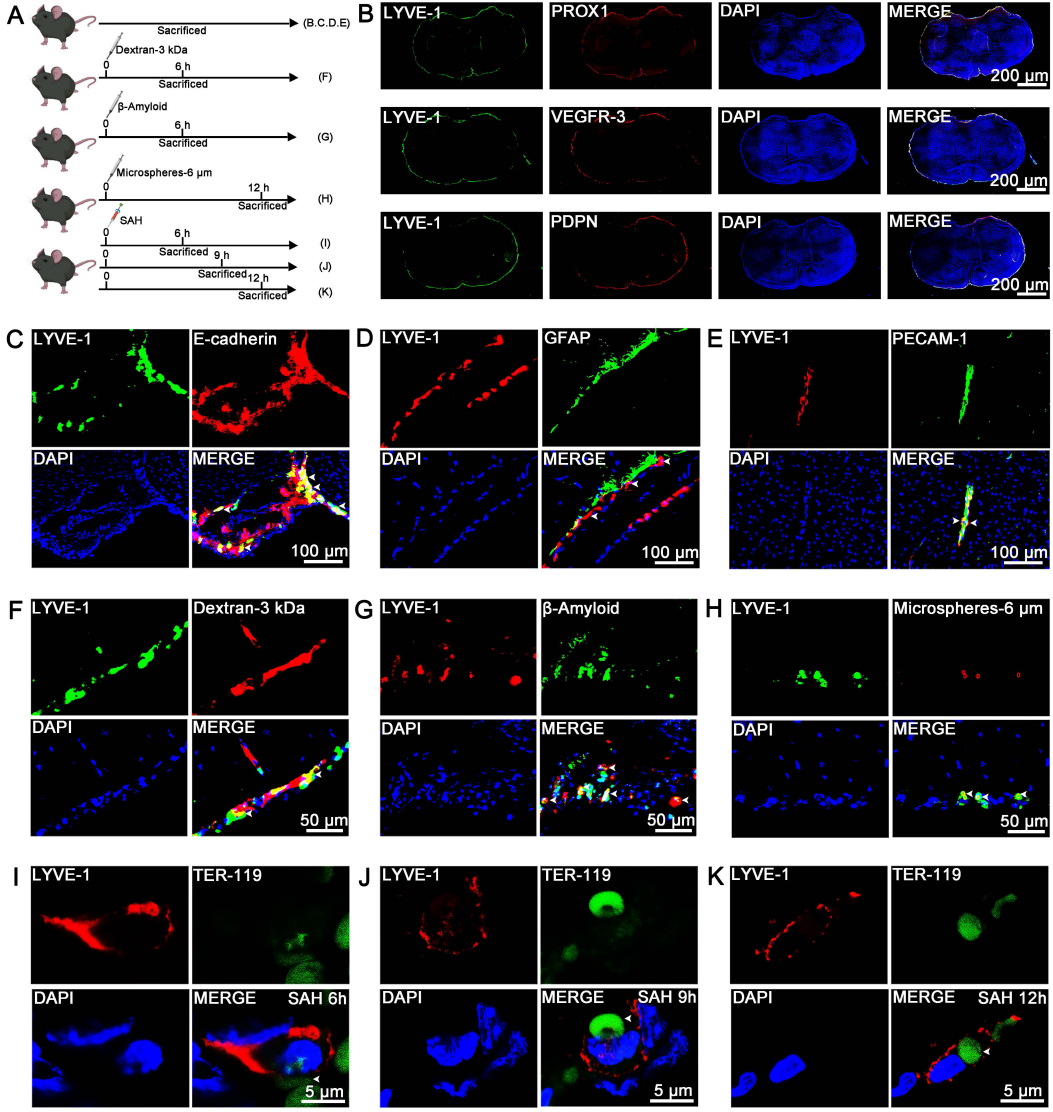
** The anatomical distribution and phagocytosis capacity of LLECs in mice. (A)** Schematic of the experimental design and procedures. **(B)** Representative immunofluorescence images showing that LYVE-1-positive cells expressed PROX1, VEGFR-3 and PDPN and encircled the exterior surface of the brain (scale bars: 200 μm). **(C)** Representative immunofluorescence images indicating that LYVE-1-positive cells were localized in the E-cadherin-positive arachnoid layer (indicated by white arrows; scale bars: 100 μm). **(D)** Representative immunofluorescence images showing that LYVE-1-positive cells adhered to the outer surface of GFAP-positive parenchymal astrocytes (indicated by white arrows; scale bars: 100 μm). **(E)** Representative immunofluorescence images revealing that LYVE-1-positive cells were peripheral to PECAM-1-positive vascular endothelial cells (indicated by white arrows; scale bars: 100 μm). **(F)** Representative immunofluorescence images showing that LYVE-1-positive cells phagocytized dextran-3 kDa (indicated by white arrows; scale bars: 50 μm). **(G)** Representative immunofluorescence images revealing that LYVE-1-positive cells phagocytized β-amyloid (indicated by white arrows; scale bars: 50 μm). **(H)** Representative immunofluorescence images showing that LYVE-1-positive cells phagocytized microspheres-6 μm (indicated by white arrows; scale bars: 50 μm). **(I-K)** Representative immunofluorescence images demonstrating that LYVE-1-positive cells phagocytized TER-119-positive erythrocytes within 6 to 12 hours post-SAH (indicated by white arrows; scale bars: 5 μm). LYVE-1, a lymphatic endothelial cell marker; PROX1, a lymphatic endothelial cell marker; VEGFR-3, a lymphatic endothelial cell marker; PDPN, a lymphatic endothelial cell marker; E-cadherin, an arachnoid layer marker; GFAP, an astrocyte marker; PECAM-1, a vascular endothelial cell marker; TER-119, an erythrocyte marker; DAPI, a nuclear marker.

Unlike the lymphatic vessels in the dura mater, LLECs are sparsely distributed and adhere to the surface of the brain 23, 48. However, the anatomical distribution of LLECs in relation to the arachnoid layer, astrocytes, vascular endothelial cells, pia mater, and perivascular spaces remains unclear. Schematic of the experimental design and procedures was shown (Figure 2A; Figure S1A; Figure S2A). Representative images revealed that lymphatic vessel endothelial HA receptor-1 (LYVE-1)-positive cells expressed prospero-related homeobox 1 (PROX1), vascular endothelial growth factor receptor-3 (VEGFR-3), and podoplanin (PDPN), and encircled the exterior surface of the brain (Figure 2B; Figure S1B). PROX1-positive, VEGFR-3-positive, and PDPN-positive cells were located within AQP-4-positive perivascular spaces and peripheral to platelet endothelial cell adhesion molecule-1 (PECAM-1)-positive vascular endothelial cells (Figure S1C, D). LYVE-1-positive cells were peripheral to PECAM-1-positive vascular endothelial cells and distinct from F4/80-positive cells (Figure S1E). Dextran-3 kDa and TER-119-positive erythrocytes were distributed at the surface of the brain and gradually decreased from 1 to 12 hours (Figure S2B, C). Additional, dextran-3 kDa were located within AQP-4-positive perivascular spaces (Figure S2D), and TER-119-positive erythrocyte were located within AQP-4-positive perivascular spaces and plectin-positive pia mater, and peripheral to PECAM-1-positive vascular endothelial cells (Figure S2E, F). Furthermore, LYVE-1-positive cells were localized to the E-cadherin-positive arachnoid layer and AQP-4-positive perivascular spaces, adhered to the outer surface of glial fibrillary astrocytic protein (GFAP)-positive parenchymal astrocyte, and peripheral to PECAM-1-positive vascular endothelial cells (Figure 2C-E, Figure S2G, H). LLECs clear macromolecules from the CSF 23, 48, suggesting a potential capacity for the autologous clearance of extravasated erythrocytes under pathophysiological conditions. At present, it is unknown whether LLECs clear erythrocytes post-SAH. After injecting dextran-3 kDa, β-amyloid, or microspheres-6 μm into the CSF, LYVE-1-positive cells phagocytized these substances (Figure 2F-H, Figure S2G). Critically, LYVE-1-positive cells also phagocytized erythrocytes within 6 to 12 hours post-SAH (Figure 2I-K, Figure S2H). The above results indicate that LLECs surround the SAS and possess the novel capacity to phagocytize dextran-3 kDa, β-amyloid, microspheres-6 μm, and erythrocytes in mice.

### Eryptosis initiates the degradation of erythrocytes in ACSF

**Figure 3:**
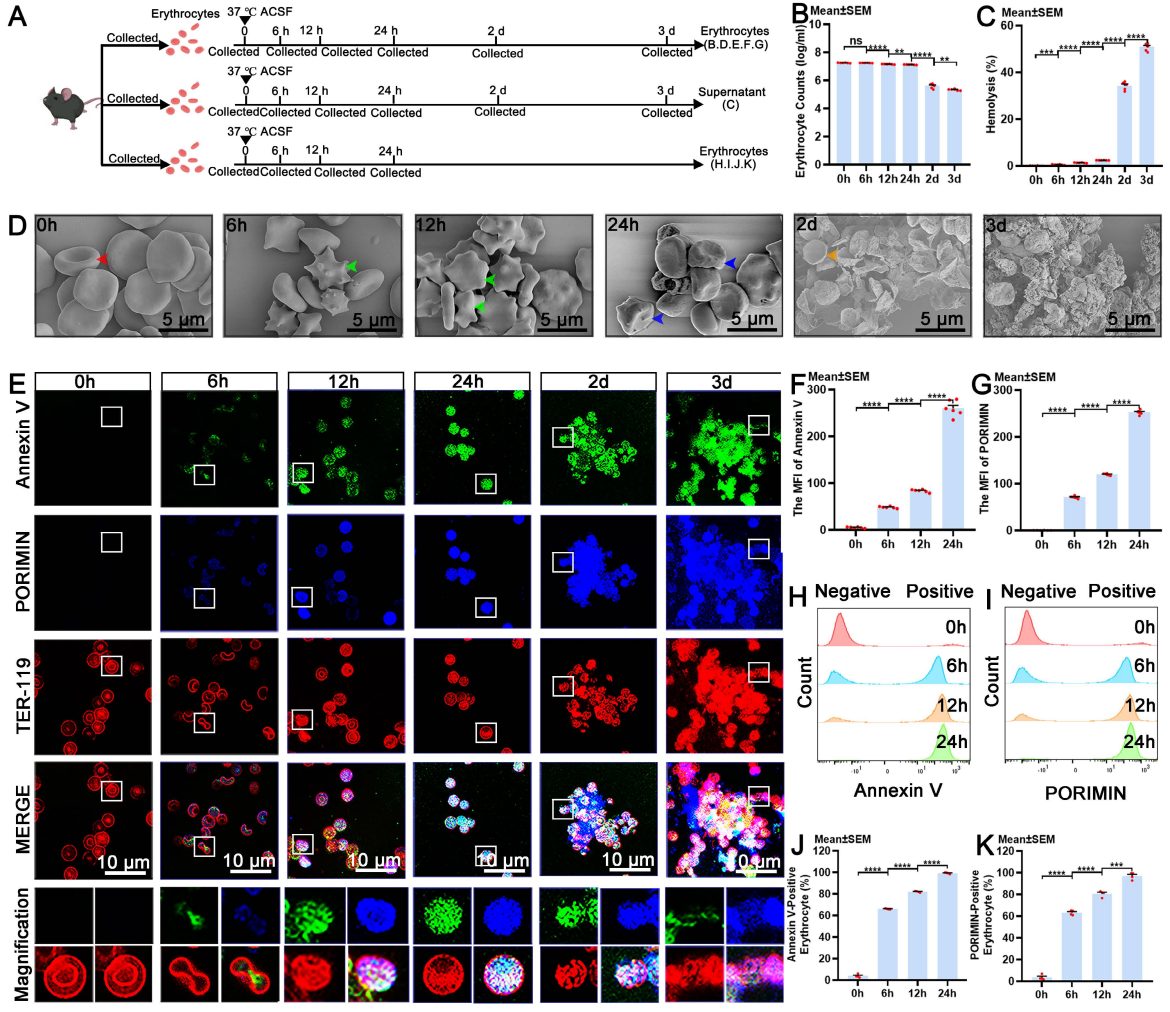
** The cellular dynamics of eryptosis, oncosis and degradation in erythrocytes. (A)** Schematic of the experimental design and procedures. **(B)** Quantification of erythrocyte counts in ACSF at various time points by counts analysis (n = 6). **(C)** Quantification of the percentage of hemolysis in ACSF at various time points by hemoglobin release detection (n = 6). **(D)** Representative scanning electron microscope images showing the morphological characteristics of erythrocytes in ACSF at various time points (indicated by color arrows; scale bars: 5 μm). **(E)** Representative immunofluorescence images showing the expression of Annexin V-positive, PORIMIN-positive and TER-119-positive erythrocytes in ACSF at various time points (scale bars: 10 μm). **(F)** Quantification of the MFI of Annexin V by immunofluorescence (n = 6). **(G)** Quantification of the MFI of PORIMIN by immunofluorescence (n = 6). **(H)** Representative flow cytometry displaying the expression of Annexin V in erythrocytes in ACSF at various time points. **(I)** Representative flow cytometry displaying the expression of PORIMIN in erythrocytes in ACSF at various time points. **(J)** Quantification of the percentage of Annexin V-positive erythrocytes by flow cytometry analysis (n = 4). **(K)** Quantification of the percentage of PORIMIN-positive erythrocytes by flow cytometry analysis (n = 4). Annexin V, an apoptosis marker; PORIMIN, an oncosis marker; TER-119, an erythrocyte marker. The data are presented as the means ± SEM; *P < 0.05, **p < 0.01, ***p < 0.005; ****p < 0.001; ns, not significant.

It is essential to investigate the pathophysiological processes that erythrocytes undergo in the CSF to elucidate the pathway for the autologous clearance of extravasated erythrocytes within the SAS. Therefore, our research focused on revealing the cellular dynamics of erythrocytes. Eryptosis is a form of programmed erythrocyte death that culminates in the elimination of erythrocytes without membrane disruption or the release of intracellular substances 17. It is characterized by phosphatidylserine (PS) exposure, membrane blebbing, and cellular shrinkage 49. Annexin V owns the ability to bind PS specifically, which makes it useful for detecting the exposure of PS 50. Additionally, oncosis is a passive form of cell death that is characterized by cellular swelling and is distinct from other cell death processes 51, 52. Pro-oncosis receptor inducing membrane injury (PORIMIN), also known as transmembrane protein 123 (TMEM123), serves as a specific marker for oncosis 53. Eryptosis triggers the clearance of erythrocyte to prevent degradation, whereas oncosis frequently results in the release of intracellular substances. Despite ongoing research, the cellular dynamics of erythrocytes in the CSF, particularly with respect to eryptosis, oncosis, and degradation, remain largely unknown. Herein, erythrocytes were incubated in ACSF at 37 °C to mimic the conditions of the SAH *in vitro.* Schematic of the experimental design and procedures was shown (Figure 3A; Figure S3A).

Our findings indicated that the number of erythrocytes significantly decreased at 12 hours and continued to decrease over time, reaching its lowest level at 3 days (Figure 3B). The release of hemoglobin from degradation began to notably increase at 6 hours and continued to increase progressively for 3 days (Figure 3C), accompanied by a gradual increase in the intensity of the color of the supernatant (Figure S3B). Representative images from scanning electron microscope revealed that intact erythrocytes possessed a biconcave disc shape at 0 hours, apoptotic erythrocytes displayed membrane blebbing and cellular shrinkage at 6 and 12 hours, and erythrocytes exhibited cellular swelling, a morphology indicative of oncotic erythrocytes at 24 hours, and degraded by 2 days (Figure 3D). Moreover, we confirmed that the expression of Annexin V and PORIMIN on erythrocytes, and the percentage of Annexin V-positive and PORIMIN-positive erythrocytes began to increase at 6 hours and then gradually increased until 24 hours (Figure 3E-K). The results of erythrocyte membrane disruption analysis revealed that the erythrocyte membrane began to be disrupted at 6 hours and continued to deteriorate until 3 days (Figure S3C-D). The results from light microscopy were consistent with those from scanning electron microscope (Figure. S3E). Erythrocyte size decreased at 6 hours and subsequently increased at 24 hours, aligning with the respective timelines of eryptosis and oncosis, respectively (Figure S3F). These results suggest that the cellular dynamic changes of erythrocytes in ACSF include membrane disruption, eryptosis, oncosis, and degradation, and eryptosis initiates degradation of erythrocytes.

### The efferocytosis of erythrocytes by LLECs

**Figure 4:**
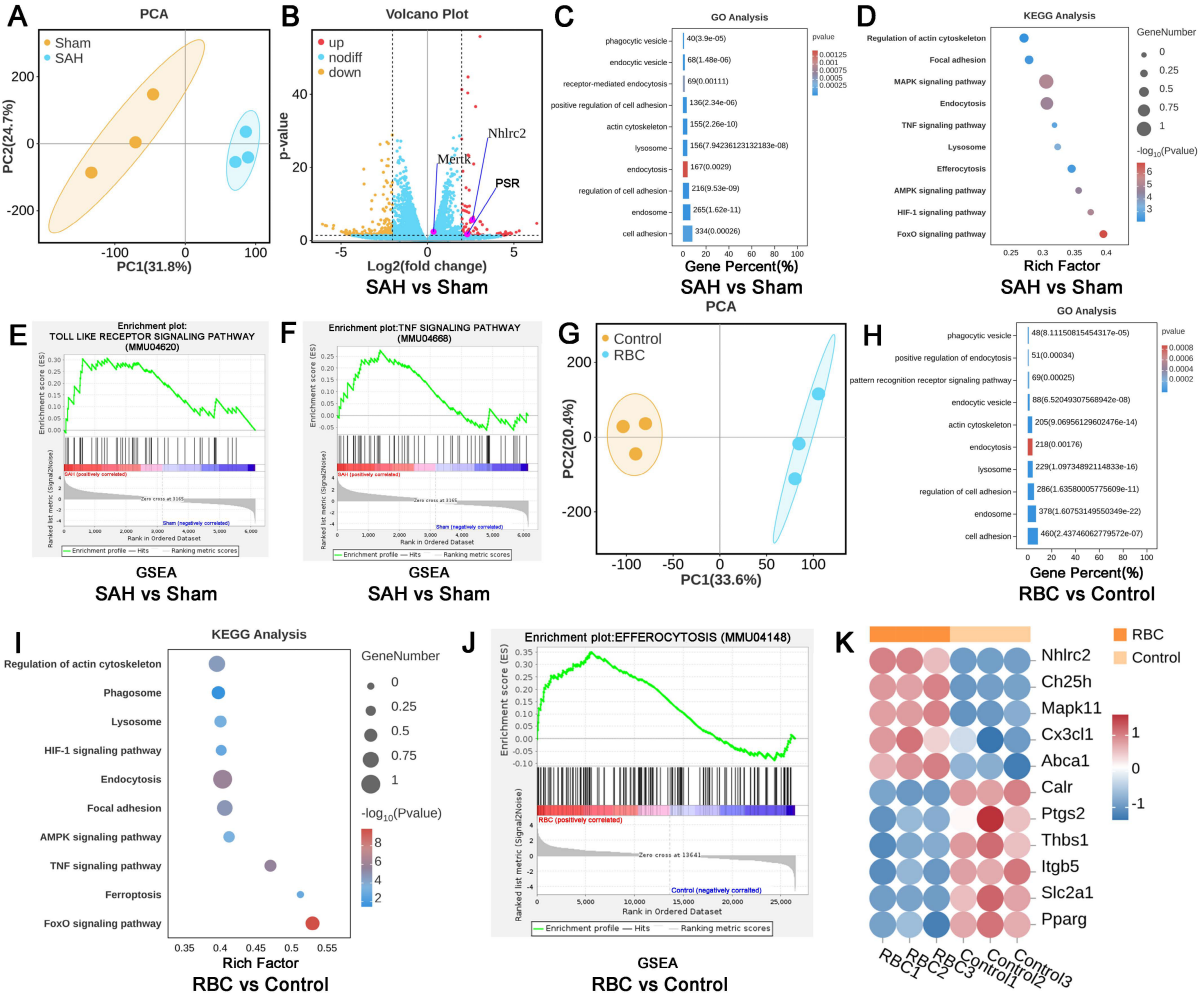
** The efferocytosis of erythrocytes by LLECs. (A)** PCA between the SAH vs sham group. **(B)** Volcano plot between the SAH vs sham group. **(C)** GO enrichment of DEGs between the SAH vs sham group. **(D)** KEGG enrichment of DEGs between the SAH vs sham group. **(E-F)** GSEA between the SAH vs sham group. **(G)** PCA between the RBC vs control group. **(H)** GO enrichment of DEGs between the RBC vs control group. **(I)** KEGG enrichment of DEGs between the RBC vs control group. **(J)** GSEA between the RBC vs control group. **(K)** DEGs analysis between the RBC vs control group in efferocytosis gene set. |log2(FC)|>2, p-value≤0.05, |NES|>1, and FDR q-value<0.25 represent differences.

To further confirm the biological processes between LLECs and erythrocytes, LLECs were obtained via magnetic-activated cell sorting post-SAH, and LLECs were collected after erythrocytes addition *in vitro* for RNA sequencing. DEGs analysis results showed that the expression of NHLRC2, Mertk, and PSR genes in the SAH group was markedly upregulated, in contrast with the sham group (Figure 4A, B). Compared with the SAH and Sham groups, GO enrichment analysis exhibited that DEGs were enriched in cell adhesion, endosome, regulation of cell adhesion, endocytosis, lysosome, actin cytoskeleton, positive regulation of cell adhesion, receptor-mediated endocytosis, endocytic vesicle, and phagocytic vesicle (Figure 4C). KEGG enrichment analysis manifested that DEGs were enriched in endocytosis, regulation of actin cytoskeleton, lysosome, TNF signaling pathway, efferocytosis, MAPK signaling pathway, focal adhesion, AMPK signaling pathway, HIF-1 signaling pathway, and FoxO signaling pathway (Figure 4D). GSEA results displayed that the Toll-like pathway and TNF pathway were upregulated in the SAH group (Figure 4E-F). Subsequently, further analysis was conducted between the RBC and control groups *in vitro* experiments (Figure 4G). GO enrichment analysis presented that DEGs were enriched in cell adhesion, endosome, lysosome, endocytosis, actin cytoskeleton, endocytic vesicle, pattern recognition receptor signaling pathway, positive regulation of endocytosis, phagocytic vesicle, and regulation of cell adhesion (Figure 4H). KEGG enrichment analysis indicated that DEGs were enriched in the FoxO signaling pathway, endocytosis, focal adhesion, AMPK signaling pathway, lysosome, TNF signaling pathway, ferroptosis, HIF-1 signaling pathway, regulation of actin cytoskeleton, and phagosome (Figure 4I). GSEA results revealed that efferocytosis was upregulated in the RBC group compared with the control group (Figure 4J). After defining the efferocytosis gene set through KEGG and performing DEGs analysis, the results showed that NHLRC2, Ch25h, Mapk11, Cx3cl1, and Abca1 genes were notably upregulated, while Calr, Ptgs2, Thbs1, Itgb5, Slc2a1, and Pparg genes were remarkably downregulated in the RBC group compared with the control group (Figure 4K). Those above analysis results indicate that LLECs undergo efferocytosis-related biological processes post-SAH.

### PS and phosphatidylserine receptor (PSR) mediate the recognition of apoptotic erythrocytes by LLECs during efferocytosis

**Figure 5:**
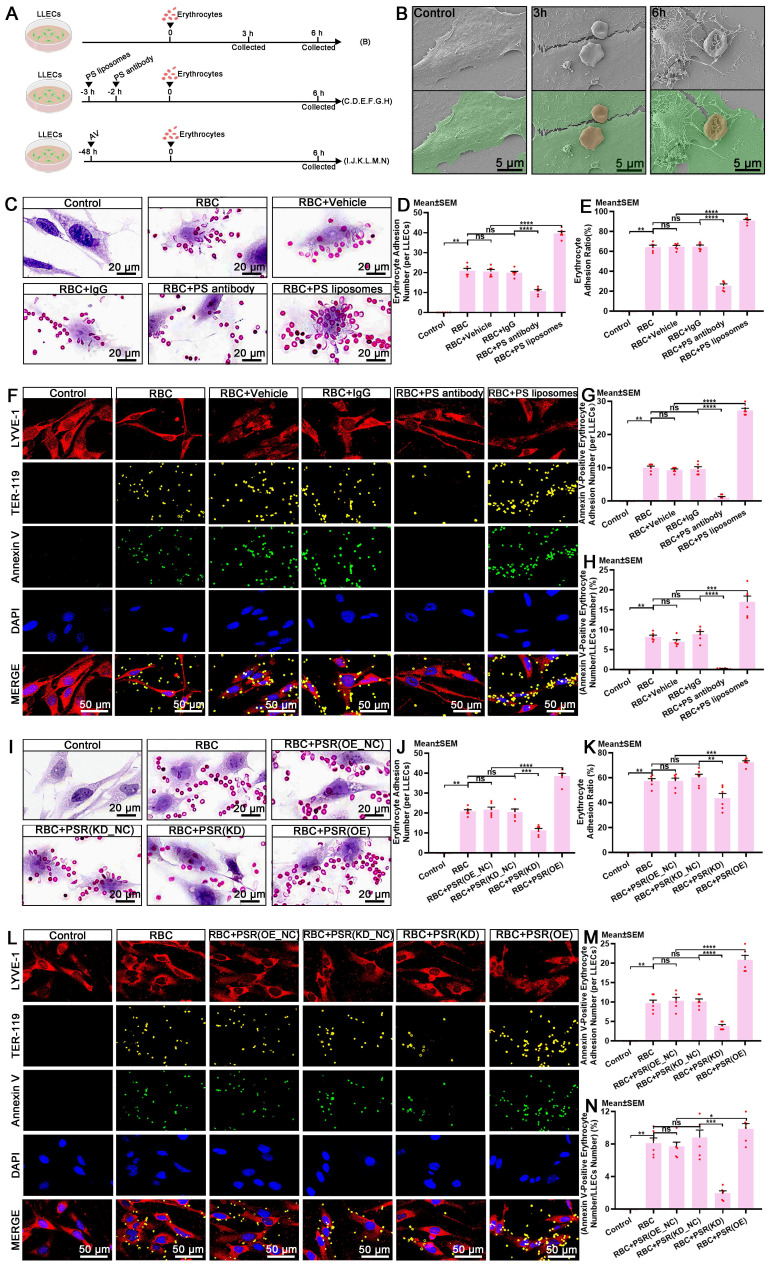
** PS and PSR mediate the recognition of apoptotic erythrocytes by LLECs during efferocytosis. (A)** Schematic of the experimental design and procedures. **(B)** Representative scanning electron microscope images depicting the adhesive interactions between LLECs and erythrocytes *in vitro* (LLECs are indicated by green pseudocolor; erythrocytes are indicated by red pseudocolor; scale bars: 5 μm). **(C)** Representative H&E staining images of LLECs and erythrocytes *in vitro* (scale bars: 20 μm). **(D)** Quantification of erythrocyte adhesion number (per LLECs) by H&E staining (n = 6). **(E)** Quantification of the percentage of erythrocyte adhesion ratio by adhesion assays (n = 6). **(F)** Representative immunofluorescence images showing the expression and adhesion status of Annexin V-positive, TER-119-positive erythrocytes and LYVE-1-positive LLECs* in vitro* (scale bars: 50 μm). **(G)** Quantification of Annexin V-positive erythrocyte adhesion number (per LLECs) by immunofluorescence staining (n = 6). **(H)** Quantification of the percentage of Annexin V-positive erythrocyte number/LLECs number by immunofluorescence staining (n = 6). **(I)** Representative H&E staining images of LLECs and erythrocytes *in vitro* (scale bars: 20 μm). **(J)** Quantification of erythrocyte adhesion number (per LLECs) by H&E staining (n = 6). **(K)** Quantification of the percentage of erythrocyte adhesion ratio by adhesion assays (n = 6). **(L)** Representative immunofluorescence images showing the number and adhesion status of Annexin V-positive, TER-119-positive erythrocytes and LYVE-1-positive LLECs *in vitro* (scale bars: 50 μm). **(M)** Quantification of Annexin V-positive erythrocyte adhesion number (per LLECs) by immunofluorescence staining (n = 6). **(N)** Quantification of the percentage of Annexin V-positive erythrocyte number/LLECs number by immunofluorescence staining (n = 6). Annexin V, an apoptosis marker; TER-119, an erythrocyte marker; LYVE-1, a lymphatic endothelial cell marker; DAPI, a nuclear marker. The data are presented as the means ± SEM; *P < 0.05, **p < 0.01, ***p < 0.005; ****p < 0.001; ns, not significant.

The discovery that eryptosis occurs in ACSF implies that the clearance of erythrocytes via efferocytosis can effectively prevent the release of degradative products and subsequent brain injury post-SAH. The exposure of PS on the surface of apoptotic erythrocytes serves as a recognition signal that instigates efferocyte-mediated efferocytosis 20, 49, 54, 55. Accumulating evidence has shown that PS and its receptors mediate the biological interactions between erythrocytes and efferocytes during efferocytosis 20, 49, 56. PSR, also known as Jumonji domain-containing protein 6 (JMJD6), is expressed on efferocytes and mediates the recognition of PS-positive erythrocytes 56-58. Presently, the mechanism underlying the recognition of erythrocytes by LLECs during efferocytosis remains incompletely understood. Schematic of the experimental design and procedures was shown (Figure 5A; Figure S4A). Representative images showed the adhesive interactions between LLECs and erythrocytes at 3 and 6 hours *in vitro* (Figure 5B). Blocking with a PS antibody notably reduced the adhesion between Annexin V-positive erythrocytes and LYVE-1-positive cells, whereas encapsulating erythrocytes with PS liposomes markedly bolstered adhesion both *in vitro* and *in vivo* (Figure 5C-H; Figure S4B, C). Moreover, PS positively regulated the expression of PSR in vitro (Figure S4D-E). Knockdown of PSR remarkably diminished its mRNA expression, while overexpression of PSR substantially enhanced its mRNA expression *in vivo* (Figure S4F). Knockdown of PSR significantly diminished adhesion between Annexin V-positive erythrocytes and LYVE-1-positive cells, while overexpression of PSR substantially enhanced adhesion *in vitro* (Figure 5I-N). These findings demonstrate that PS and PSR facilitate the recognition of apoptotic erythrocytes by LLECs during efferocytosis.

### NHL repeat-containing 2 (NHLRC2) promotes the efferocytosis of erythrocytes by LLECs

**Figure 6:**
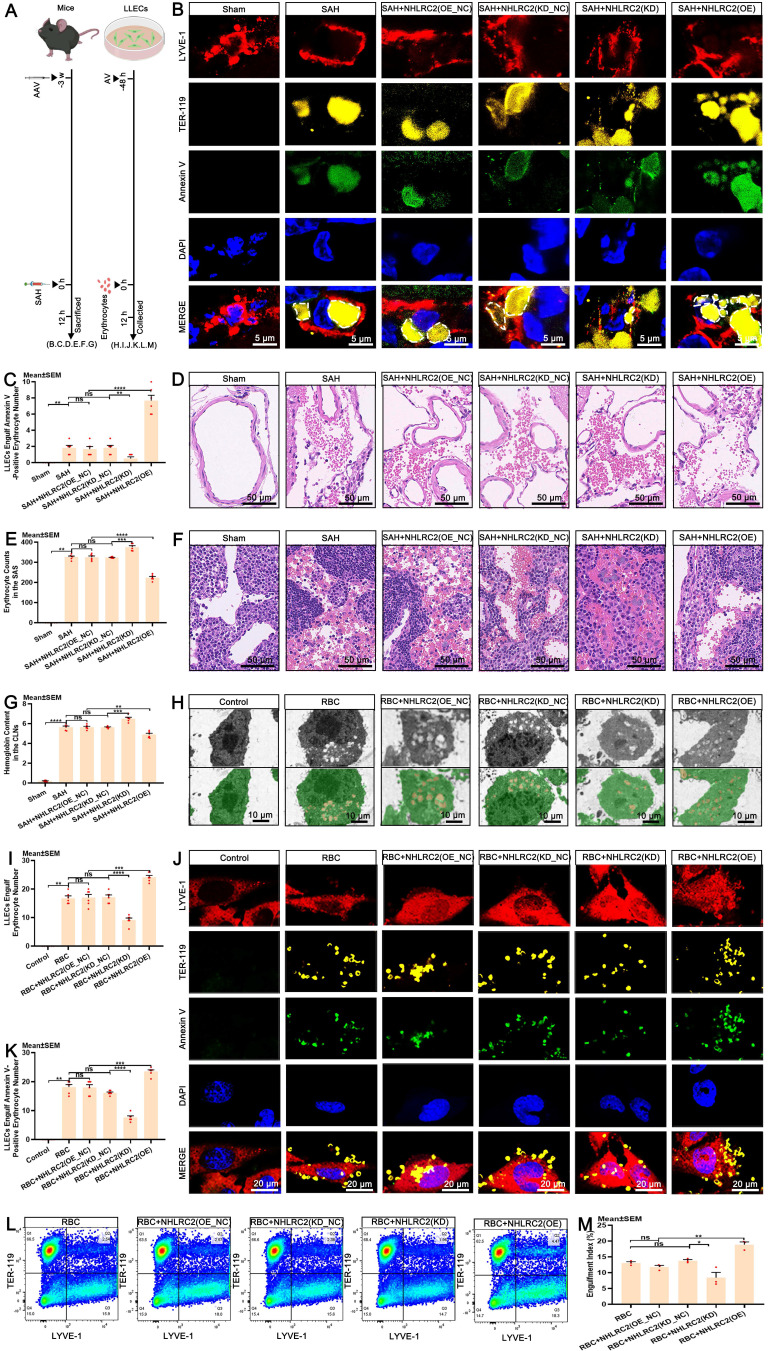
** NHLRC2 governs the efferocytosis of erythrocytes by LLECs. (A)** Schematic of the experimental design and procedures. **(B)** Representative immunofluorescence images showing the expression and engulfment status of Annexin V-positive, TER-119-positive erythrocytes and LYVE-1-positive LLECs *in vivo* (scale bars: 5 μm). **(C)** Quantification of the number of LLECs engulfing Annexin V-positive erythrocyte by immunofluorescence staining (n = 6). **(D)** Representative H&E staining images showing the SAS (scale bars: 50 μm). **(E)** Quantification of erythrocyte counts in the SAS by H&E staining (n = 6). **(F)** Representative H&E staining images showing the CLNs (scale bars: 50 μm). **(G)** Quantification of the hemoglobin content in the CLNs by hemoglobin content detection (n = 6). **(H)** Representative TEM images depicting the engulfment interactions between LLECs and erythrocytes *in vitro* (LLECs are indicated by green pseudocolor; erythrocytes are indicated by red pseudocolor; scale bars: 10 μm). **(I)** Quantification of the number of erythrocytes LLECs engulfing by TEM (n = 6). **(J)** Representative immunofluorescence images showing the expression and engulfment status of Annexin V-positive, TER-119-positive erythrocytes and LYVE-1-positive LLECs* in vitro* (scale bars: 20 μm). **(K)** Quantification of the number of Annexin V-positive erythrocyte LLECs engulfing by immunofluorescence staining (n = 6). **(L)** Representative flow cytometry plots showing the expression of TER-119 and LYVE-1 *in vitro.* **(M)** Quantification of the percentage of engulfment index by flow cytometry analysis (n = 3). Annexin V, an apoptosis marker; TER-119, an erythrocyte marker; LYVE-1, a lymphatic endothelial cell marker; DAPI, a nuclear marker. The data are presented as the means ± SEM; *P < 0.05, **p < 0.01, ***p < 0.005; ****p < 0.001; ns, not significant.

Upon recognizing apoptotic cells, efferocytes execute their clearance capacity by engulfing them 18, 59, 60. Importantly, the engulfment of apoptotic erythrocytes by LLECs achieves the complete autologous clearance of extravasated erythrocytes post-SAH. However, the specific regulatory targets that govern this process are not yet fully understood. NHLRC2 has been proposed to be a potent regulator in efferocytes 61, 62. The regulatory function of NHLRC2 in the efferocytosis of erythrocytes by LLECs remains largely unexplored. Schematic of the experimental design and procedures was shown (Figure 6A; Figure S5A). Time-lapse images revealed the dynamic process by which LLECs engulfed erythrocytes between 6 to 9.5 hours post-co-incubation (Figure S5B; Video S1). Ionomycin treatment significantly increased the number of Annexin V-positive erythrocytes (Figure S5C-D) and substantially boosted the engulfment of apoptotic erythrocytes by LLECs (Figure S5E-F). The expression of NHLRC2 in LLECs gradually increased from 6 to 12 hours after erythrocyte addition *in vitro* (Figure S5G-H). Knockdown of PSR remarkably suppressed the mRNA expression of NHLRC2, while overexpression of PSR substantially enhanced the mRNA expression of NHLRC2 *in vitro* (Figure S5I)*.* The efferocytosis gene set of DEGs analysis results displayed that the NHLRC2 and Dusp4 genes were remarkably downregulated, while the Bsg gene was notably upregulated in the RBC+NHLRC2(KD) group compared with the RBC group (Figure S5J).

Knockdown of NHLRC2 remarkably diminished its mRNA expression, while overexpression of NHLRC2 substantially increased its mRNA expression *in vivo* (Figure S5K). Subsequently, we verified the regulatory function of NHLRC2 in the efferocytosis of erythrocytes by LLECs both *in vivo* and *in vitro*. The results indicated that knockdown of NHLRC2 notably inhibited the engulfment of Annexin V-positive erythrocytes by LYVE-1-positive cells and markedly increased the erythrocyte counts in the SAS and the hemoglobin content in the brain and CLNs, whereas overexpression of NHLRC2 apparently enhanced the engulfment of Annexin V-positive erythrocytes by LYVE-1-positive cells and obviously reduced the erythrocyte count in the SAS and the hemoglobin content in the brain and CLNs post-SAH (Figure 6B-G, Figure S5L). Our findings also demonstrated that knockdown of NHLRC2 noticeably suppressed the engulfment of Annexin V-positive erythrocytes by LYVE-1-positive cells, while overexpression of NHLRC2 significantly amplified this process *in vitro* (Figure 6H-M). The above evidence shows that NHLRC2 promotes the efferocytosis of erythrocytes by LLECs.

### NHLRC2 regulates LLECs-mediated neuroprotection post-SAH

**Figure 7:**
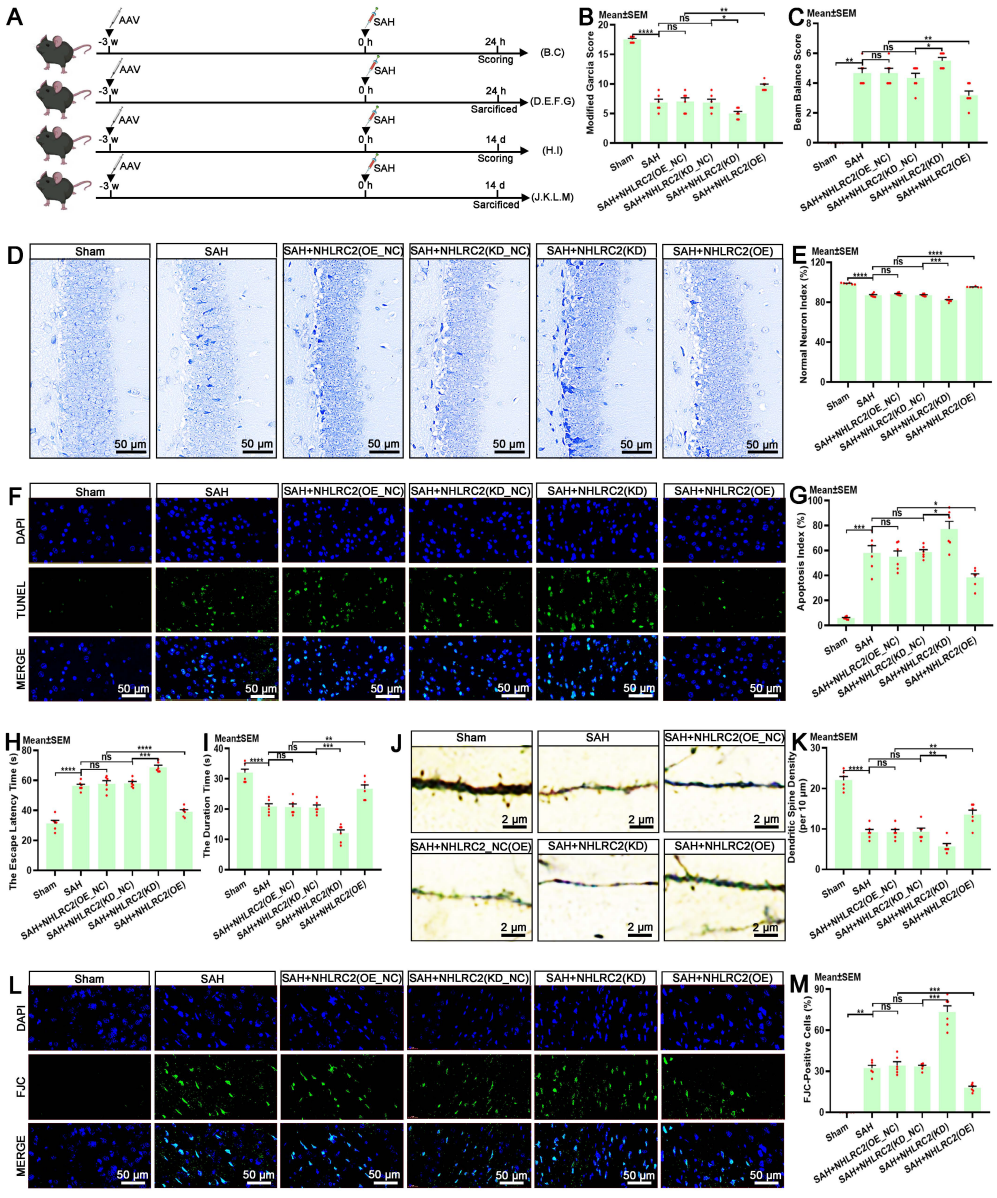
** NHLRC2 influences neurological functions and neurostructural integrity post-SAH. (A)** Schematic of the experimental design and procedures. **(B)** Evaluation of short-term neurological function with the modified Garcia score (n = 6). **(C)** Evaluation of short-term neurobehavioral effects with the beam balance test (n = 6). **(D)** Representative Nissl staining images indicating the functional state of neurons in the hippocampus post-SAH (scale bars: 50 μm). **(E)** Quantification of the percentage of normal neurons index by Nissl staining (n = 6). **(F)** Representative TUNEL staining images revealing apoptotic cells in the temporal lobe cortex post-SAH (scale bars: 50 μm). **(G)** Quantification of apoptosis index by TUNEL staining (n = 6). **(H)** Evaluation of long-term neurological function with the escape latency time by the Barnes maze test (n = 6). **(I)** Evaluation of long-term neurobehavioral effects with the duration time by the rotarod test (n = 6). **(J)** Representative Golgi staining images demonstrating the synaptic function of neurons in the temporal lobe cortex post-SAH (scale bars: 2 μm). **(K)** Quantification of dendritic spine density (per 10 μm) by Golgi staining (n = 6). **(L)** Representative FJC staining images showing degenerating neurons in the temporal lobe cortex post-SAH (scale bars: 50 μm). **(M)** Quantification of the percentage of FJC-positive cells by FJC staining (n = 6). DAPI, a nuclear marker. The data are presented as the means ± SEM; *P < 0.05, **p < 0.01, ***p < 0.005; ****p < 0.001; ns, not significant.

**Figure 8:**
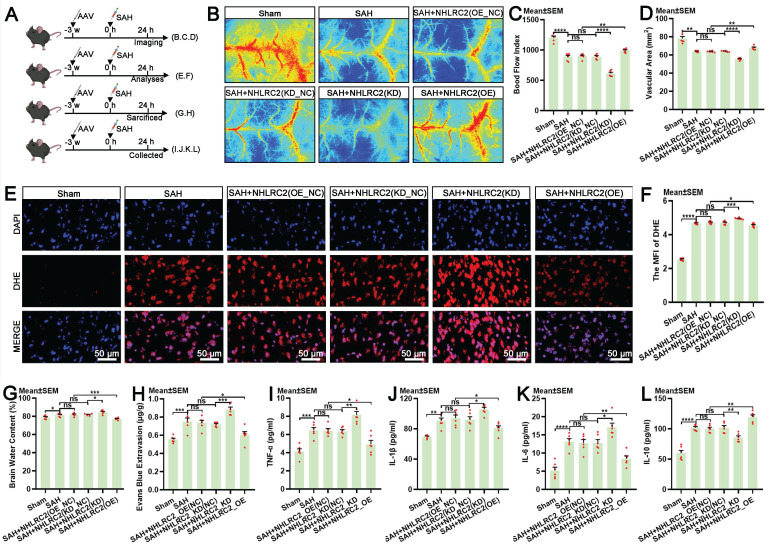
** NHLRC2 impacts pathological consequences post-SAH. (A)** Schematic of the experimental design and procedures. **(B)** Representative laser speckle contrast image reflecting brain blood perfusion post-SAH. **(C)** Quantification of the blood flow index by laser speckle contrast imaging (n = 6). **(D)** Quantification of the vascular area by laser speckle contrast imaging (n = 6). **(E)** Representative DHE staining images showing the levels of ROS in the temporal lobe cortex post-SAH (scale bars: 50 μm). **(F)** Quantification of the MFI of DHE by ROS detection (n = 6). **(G)** Quantification of the percentage of brain water content post-SAH (n = 6). **(H)** Quantification of the concentration of Evans blue extravasation in blood-brain barrier integrity detection post-SAH (n = 6). **(I-L)** Quantification of the concentrations of TNF-α, IL-1β, IL-6, and IL-10 in the serum by ELISA post-SAH (n = 6). DAPI, a nuclear marker. The data are presented as the means ± SEM; *P < 0.05, **p < 0.01, ***p < 0.005; ****p < 0.001; ns, not significant.

The autologous clearance of extravasated erythrocytes via efferocytosis exerts neuroprotection against brain injury post-SAH. Hence, in this study, we thoroughly explored the role of NHLRC2 in LLECs-mediated neuroprotection, highlighting its significance. Schematic of the experimental design and procedures was shown (Figure 7A). After specific overexpression or knockdown of NHLRC2 in LLECs, the evaluation of short-term neurological functions and neurostructural integrity post-SAH revealed that the knockdown of NHLRC2 significantly reduced the modified Garcia score (Figure 7B) and percentage of normal neurons index (Figure 7D-E), and noticeably increased the beam balance score (Figure 7C) and percentage of TUNEL-positive cells (Figure 7F-G), whereas the overexpression of NHLRC2 had the opposite effects. The evaluation of long-term neurological functions and neurostructural integrity post-SAH demonstrated that the knockdown of NHLRC2 substantially increased the escape latency time to find the escape box (Figure 7H) and percentage of FJC-positive cells (Figure 7L, M), and markedly decreased the duration time on the rod (Figure 7I) and dendritic spine density (Figure 7J-K), while the overexpression of NHLRC2 led to the opposite outcomes. Schematic of the experimental design and procedures was shown (Figure 8A). The evaluation of pathological consequences post-SAH revealed that NHLRC2 knockdown greatly reduced the blood flow index (Figure 8B-C), the vascular area (Figure 8D), and the concentrations of IL-10(Figure 8L) and substantially increased the expression of dihydroethidium (DHE) (Figure 8E-F), the percentage of brain water content (Figure 8G), the Evans blue extravasation (Figure 8H), and the concentration of TNF-α, IL-1β, and IL-6 (Figure 8I-K). Conversely, the overexpression of NHLRC2 had the opposite effects. In summary, the above findings prove that NHLRC2 regulates in LLECs-mediated neuroprotection via improving neurological functions, maintaining neurostructural integrity and alleviating neuropathological consequences post-SAH.

## Discussion

This study primarily investigated the efferocytosis of erythrocytes by LLECs and its contributions post-SAH. By using *in vitro* and* in vivo* experimental approaches, we obtained the following findings: (1) LLECs surround SAS and phagocytize dextran-3 kDa, β-amyloid, microspheres-6 μm, and erythrocytes. (2) Eryptosis initiates degradation of erythrocytes in ACSF. (3) PS and PSR mediate the recognition of apoptotic erythrocytes by LLECs during efferocytosis. (4) NHLRC2 promotes the efferocytosis of erythrocytes by LLECs. (5) NHLRC2 regulates LLECs-mediated neuroprotection post-SAH. Therefore, we elucidated the efferocytosis of erythrocytes by LLECs and subsequently neuroprotection post-SAH.

The intracranial lymphatic system, which includes the glymphatic system and meningeal lymphatic vessels, is pivotal for clearing waste substances in the CSF 63, 64. LLECs, a recently discovered cellular subpopulation, are associated with the intracranial lymphatic system, but their functions are not yet fully understood 23. The anatomical distribution of LLECs confers a spatial advantage that enables direct clearance of erythrocytes within the SAS. Our findings revealed that LLECs surround the SAS and phagocytize dextran-3 kDa, β-amyloid, and microspheres-6 μm. Critically, for the first time, we observed the direct clearance of erythrocytes by LLECs post-SAH. This intriguing discovery inspired us to further investigate the pathway underlying the clearance of erythrocytes by LLECs. Erythrocytes have been reported to undergo eryptosis following intracerebral hemorrhage 21, but the pathophysiological processes of erythrocytes post-SAH have yet to be confirmed. Therefore, we concentrated on the cellular dynamic events that occur in erythrocytes and thoroughly investigated membrane disruption, eryptosis, oncosis, and degradation. Concurrent with membrane disruption, erythrocytes undergo eryptosis after 6 hours in ACSF, with apoptotic erythrocytes escaping clearance and subsequently succumbing to degradation. Notably, prompt clearance of apoptotic erythrocytes protects against brain injury stemming from subsequent degradative products post-SAH. Based on this premise, we further speculate that apoptotic erythrocytes are cleared via efferocytosis post-SAH, a pathway that makes the autologous clearance of extravasated erythrocytes feasible.

Efferocytosis enables the clearance of apoptotic cells 18. RNA sequencing analysis results indicate that LLECs undergo efferocytosis-related biological processes post-SAH. The primary LLECs that we developed offer a robust platform for investigating the efferocytosis of erythrocytes by LLECs 24. Efferocytosis involves the processes of recognition and engulfment 18. The recognition of apoptotic erythrocytes by LLECs, coupled with the engulfment of apoptotic erythrocytes by LLECs, implied that LLECs clear erythrocytes via efferocytosis. However, the regulatory targets governing the clearance of erythrocytes by LLECs via efferocytosis remain unexplored. Previous research demonstrated that the exposure of PS on apoptotic erythrocytes is a pivotal signal for recognition 20, 49, 54, 55, and PSR acted as the specific receptor for recognizing PS 56-58. After blocking erythrocytes with a PS antibody, encapsulating erythrocytes with PS liposomes, and either overexpressing or knocking down PSR in LLECs, we revealed the key roles of PS and PSR in the recognition process, as well as the positive regulatory effect of PS on PSR. Additionally, NHLRC2 was shown to govern the efferocytosis of erythrocytes by LLECs, consistent with findings obtained in macrophages 61. The efferocytosis of erythrocytes by LLECs occurred within 12 hours post-SAH, during which the erythrocytes had not yet released degradative products. Accordingly, LLECs possess the capacity to promptly clear erythrocytes via efferocytosis. The regulable pathway identified by this work also provide evidence for the development of strategies for modulating efferocytosis.

Efferocytosis serves to terminate inflammatory responses, promote self-tolerance, and activate proresolution pathways 65. Given the regulatory function of NHLRC2 in efferocytosis of erythrocytes by LLECs, its substantial impact on post-SAH attracted our attention. The evaluation indicators post-SAH are primarily related to neurological functions, neurostructural integrity, and pathological consequences. We concluded that these indicators improved following AAV-mediated overexpression of NHLRC2 in mouse LLECs, which demonstrated the efficiency of LLECs in the efferocytosis of erythrocytes. Hence, this work explored therapeutic targets associated with LLECs-mediated neuroprotection, which holds great significance.

This pioneering investigation confirmed that LLECs act as efferocytes, performing a vital scavenging function post-SAH, and experiments utilizing primary LLECs were conducted to elucidate the mechanisms of efferocytosis *in vitro*. The early occurrence of eryptosis within the first 6 hours in ACSF is a pivotal discovery for further investigating efferocytosis of erythrocytes by LLECs. Additionally, NHLRC2 promoted the efferocytosis of erythrocytes by LLECs. The evidence suggests that NHLRC2 possibly influences the efferocytosis process through Dusp4 or Bsg. These results warrant further investigation in our subsequent studies. The ability of NHLRC2 to regulate LLECs-mediated neuroprotection via the efferocytosis of erythrocytes represents a seminal discovery of this study. Our findings diverge from those reported in previous studies, which implicated phagocytosis and drainage as erythrocytes clearance pathways post-SAH. LLECs utilize a prompt, efficient and regulable pathway to facilitate the autologous clearance of extravasated erythrocytes.

This study also has several limitations. First, we established an SAH animal model to simulate SAH in humans, but the translatability of these findings to SAH patients remains unclear. We incubated erythrocytes in ACSF to mimic the conditions of the SAH *in vitro*, and we acknowledge that this approach may lack certain influencing factors present *in vivo*. In addition, efferocytosis is not a mechanism for the specific clearance of erythrocytes, and the associated targets may also influence other pathways. The present study also did not explore the potential impact of NHLRC2 on other biological functions of LLECs.

Potential strategies to augment efferocytosis include both pharmacological interventions and genetic modulation. Furthermore, the analysis of large-scale bioinformatics data is pivotal for an in-depth understanding of efferocytosis. Understanding the biological processes that follow the efferocytosis of erythrocytes by LLECs is imperative for improving our comprehension of the influence of erythrocytes on LLECs. For instance, the lysosomal digestion process following engulfment warrants validation in our future research. This study investigated the pathway for the autologous clearance of extravasated erythrocytes post-SAH, and the potential capacity of LLECs to clear degradative products also merits investigation.

## Conclusion

In conclusion, we elucidated the efferocytosis of erythrocytes by LLECs and subsequently neuroprotection post-SAH, which is positively regulated by NHLRC2. These findings highlight a prompt, efficient, and regulable pathway for the autologous clearance of extravasated erythrocytes to reveal the potential application for the sentinel function of LLEC-mediated efferocytosis and represent a promising therapeutic approach against brain injury post- SAH.

## Supplementary Material

Supplementary figures and video legend.

Supplementary video.

## Abbreviations

AAV: adeno-associated virus
AV: adenovirus
ACSF: artificial cerebrospinal fluid
AQP-4: aquaporin-4
CSF: cerebrospinal fluid
CLNs: cervical lymph nodes
DAPI: 4,6-diamidino-2-phenylindole
DHE: dihydroethidium
VEGFR-3: endothelial growth factor receptor-3
ELISA: enzyme-linked immunosorbent assay
F4/80: macrophage differentiation antigen
FJC: fluoro-Jade C
GFAP: glial fibrillary astrocytic protein
H&E: hematoxylin and eosin
IL: interleukin
JMJD6: jumonji domain-containing protein 6
LDH: lactate dehydrogenase
LLECs: leptomeningeal lymphatic endothelial cells
LYVE-1: lymphatic vessel endothelial HA receptor-1
MFI: mean fluorescence intensity
NHLRC2: NHL repeat-containing 2
ANOVA: one-way analysis of variance
OD: optical density
PS: phosphatidylserine
PSR: phosphatidylserine receptor
PECAM-1: platelet endothelial cell adhesion molecule-1
PDPN: podoplanin
PORIMIN: pro-oncosis receptor inducing membrane injury
PROX1: prospero-related homeobox 1
ROS: reactive oxygen species
RBC: red blood cell
RT-qPCR: reverse transcription-quantitative polymerase chain reaction
SEM: standard error of the mean
SAH: subarachnoid hemorrhage
SAS: subarachnoid space
TUNEL: terminal deoxynucleotidyl transferase-mediated dUTP nick-end labeling
TMEM123: transmembrane protein 123
TEM: transmission electron microscope
TNF-α: tumor necrosis factor-alpha
