# Supplementary material for: The sentinel against brain injury post-subarachnoid hemorrhage: efferocytosis of erythrocytes by leptomeningeal lymphatic endothelial cells
Source: Theranostics. 2025 Jan 20;15(6):2487–509. doi: 10.7150/thno.103701 (PMC11840724; doi:10.7150/thno.103701)
Supplement: Supplementary file 1 — Supplementary figures and video legend. [file thnov15p2487s1.pdf]

Supplemental Data

The sentinel against brain injury post-subarachnoid hemorrhage: efferocytosis of erythrocytes by leptomeningeal lymphatic endothelial cells

Hong-Ji Deng[1], Yun-Huo Xu[1], Kun Wu[2], Yun-Cong Li[1], Yong-Jin Zhang[1,3], Han-Fu Yu[1], Chong Li[1], Dan Xu[4], Fei Wang[1,*]

1. Department of Neurosurgery, The First Affiliated Hospital of Kunming Medical University, Kunming, China.

2. Department of Clinical Laboratory, The First Affiliated Hospital of Kunming Medical University, Kunming, China.

3. Clinical Medical Research Center, The First Affiliated Hospital of Kunming Medical University, Kunming, China.

4. Department of Dermatology, The First Affiliated Hospital of Kunming Medical University, Kunming, China.

*Corresponding author: Fei Wang; E-mail: wangfei@kmmu.edu.cn; Department of Neurosurgery, The First Affiliated Hospital of Kunming Medical University, Kunming, China; 295 Xichang Road, Kunming, Yunnan, 650032, China.

Contents

Supplemental Figure 1

Supplemental Figure 2

Supplemental Figure 3

Supplemental Figure 4

Supplemental Figure 5

Video S1

Supplemental Figure 1

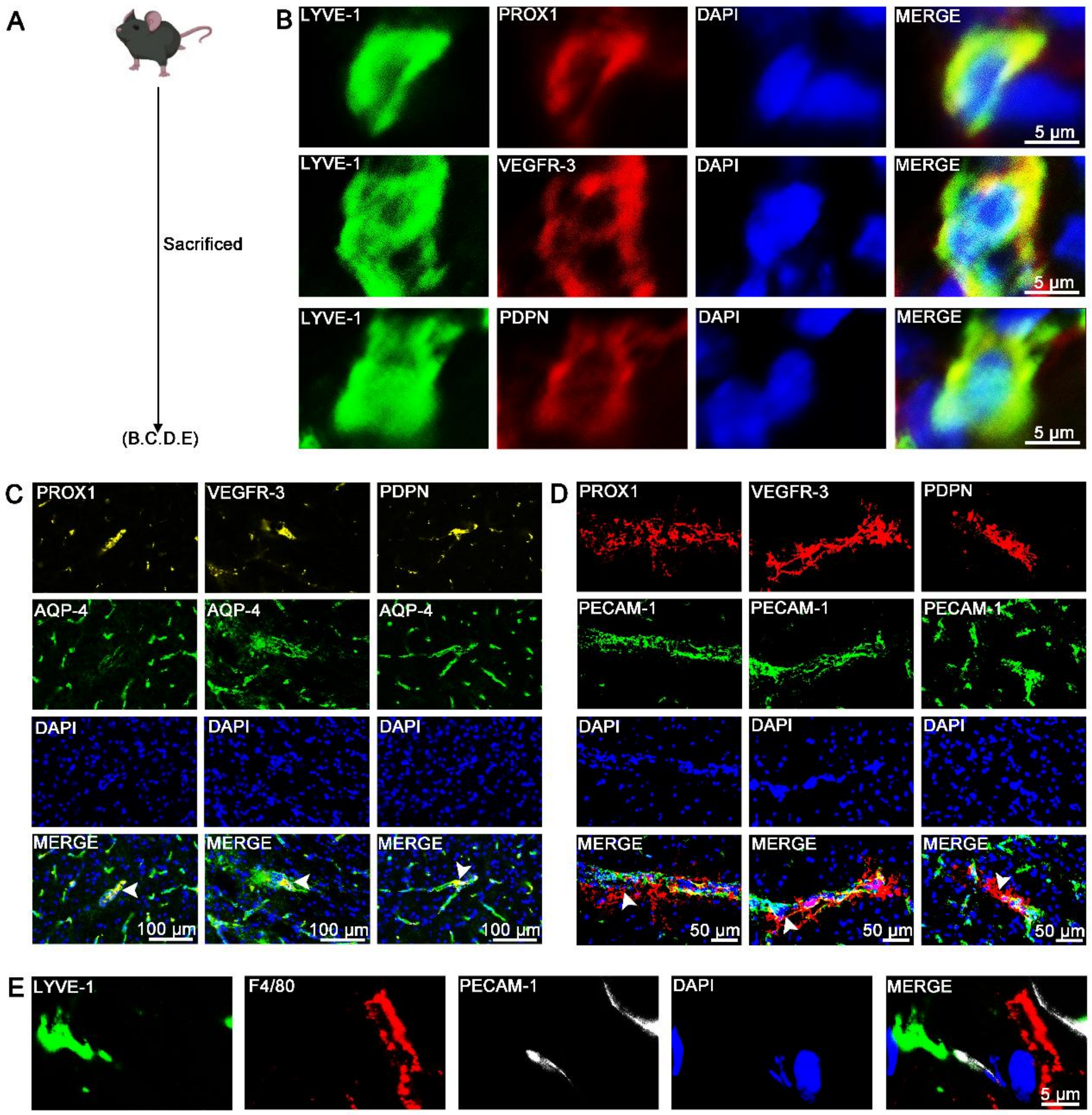

Figure S1. The identification of LLECs in mice. (A) Schematic of the experimental design and procedures. **(B)** Representative immunofluorescence images showing LYVE-1-positive cells expressed PROX1, VEGFR-3 and PDPN *in vivo* (scale bars: 5 µm). **(C)** Representative immunofluorescence images showing that PROX1-positive, VEGFR-3-positive, and PDPN-positive cells were located within AQP-4-positive perivascular spaces *in vivo* (indicated by white arrows; scale bars: 100 µm). **(D)** Representative immunofluorescence images revealing that PROX1-positive, VEGFR-3-positive, and PDPN-positive cells were peripheral to PECAM-1-positive vascular endothelial cells *in vivo* (indicated by white arrows; scale bars: 50 µm). **(E)** Representative immunofluorescence images showing LYVE-1-positive cells were peripheral to PECAM-1-positive vascular endothelial cells and distinct from F4/80-positive cells *in vivo* (scale bars: 5 µm). LYVE-1, a lymphatic endothelial cell marker; PROX1, a lymphatic endothelial cell marker; VEGFR-3, a lymphatic endothelial cell marker; PDPN, a lymphatic endothelial cell marker; AQP-4, a perivascular spaces

marker; PECAM-1, a vascular endothelial cell marker; F4/80, a macrophage marker; DAPI, a nuclear marker.

Supplemental Figure 2

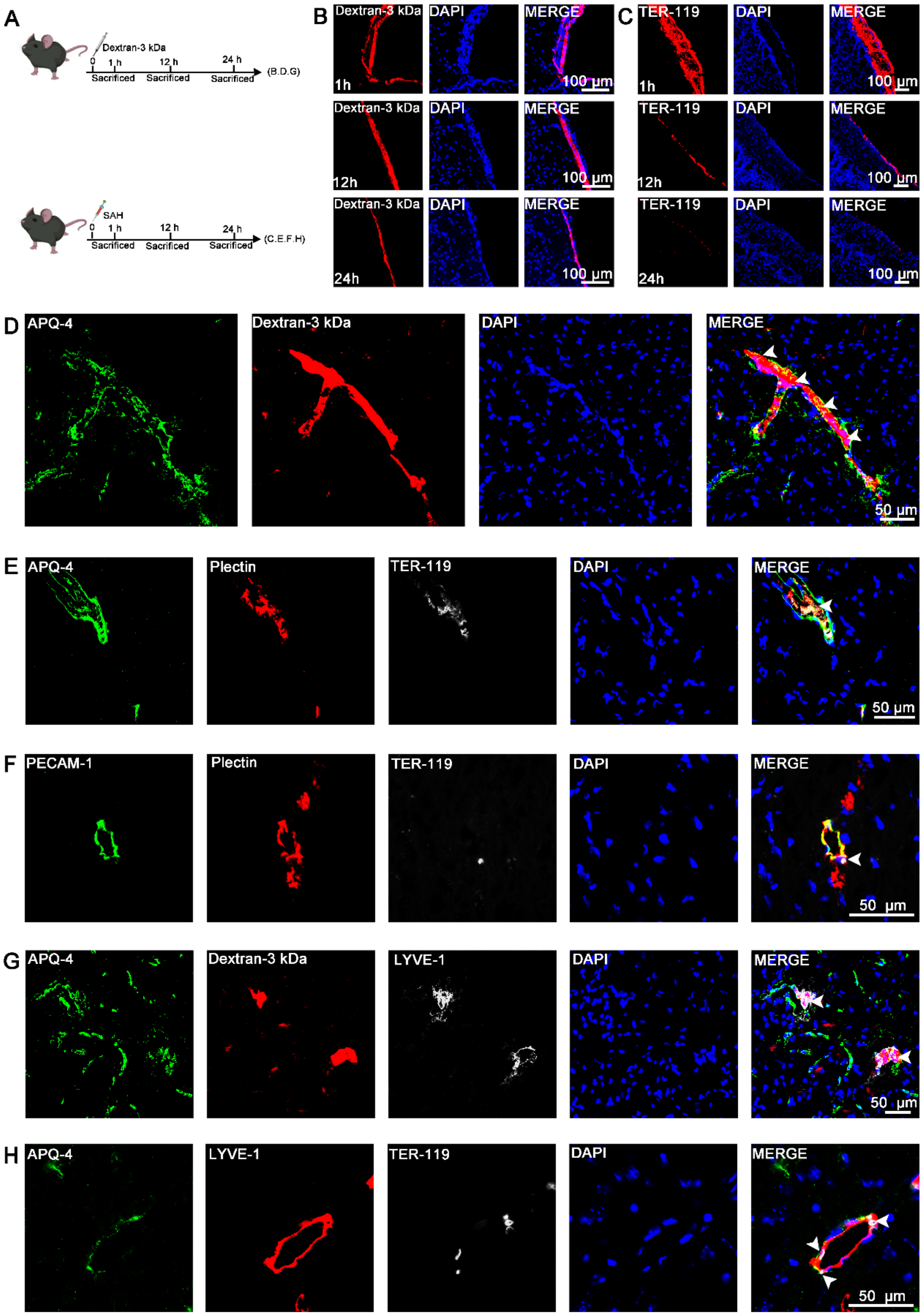

Figure S2. The distribution of dextran-3 kDa and erythrocytes in mice. (A) Schematic of the experimental design and procedures. **(B)** Representative immunofluorescence images showing the distribution of dextran-3 kDa at the surface of the brain after 1 hour, 12 hours and 24 hours *in vivo* (scale bars: 100 μm). **(C)** Representative immunofluorescence images showing the distribution of TER-119-

positive erythrocytes at the surface of the brain after 1 hour, 12 hours and 24 hours *in vivo* (scale bars: 100 μm). **(D)** Representative immunofluorescence images showing that dextran-3 kDa were located within AQP-4-positive perivascular spaces *in vivo* (indicated by white arrows; scale bars: 50 μm). **(E)** Representative immunofluorescence images showing that TER-119-positive erythrocyte were located within AQP-4-positive perivascular spaces and plectin-positive pia mater *in vivo* (indicated by white arrows; scale bars: 50 μm). **(F)** Representative immunofluorescence images showing that TER-119-positive erythrocytes were peripheral to PECAM-1-positive vascular endothelial cells and plectin-positive pia mater *in vivo* (indicated by white arrows; scale bars: 50 μm). **(G)** Representative immunofluorescence images showing that LYVE-1-positive cells were located within AQP-4-positive perivascular spaces and phagocytized dextran-3 kDa (indicated by white arrows; scale bars: 50 μm). **(H)** Representative immunofluorescence images showing that LYVE-1-positive cells were located within AQP-4-positive perivascular spaces and phagocytized TER-119-positive erythrocytes (indicated by white arrows; scale bars: 50 μm). LYVE-1, a lymphatic endothelial cell marker; TER-119, an erythrocyte marker; AQP-4, a perivascular spaces marker; PECAM-1, a vascular endothelial cell marker; Plectin, a pia mater maker; DAPI, a nuclear marker.

Supplemental Figure 3

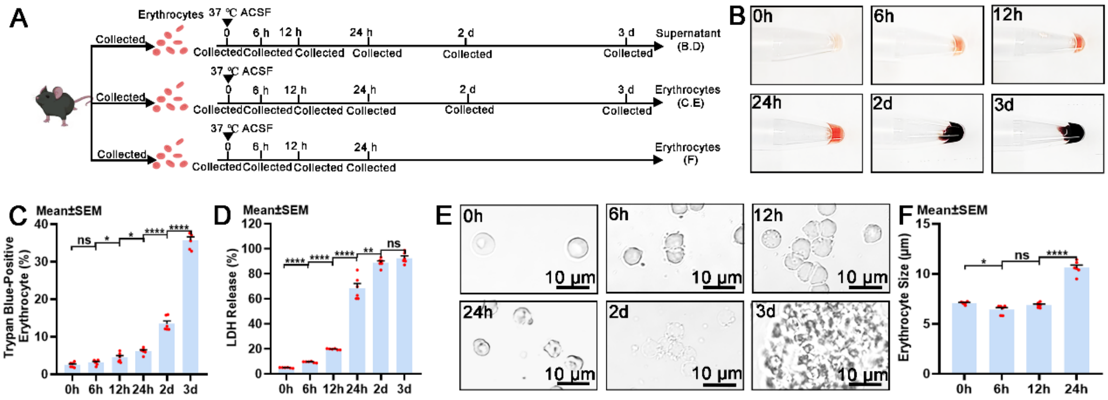

Figure S3. The cellular dynamics of membrane disruption in erythrocytes in ACSF. (A) Schematic of the experimental design and procedures. **(B)** Observation of the supernatant of erythrocytes in ACSF. **(C)** Quantification of the percentage of trypan blue-positive erythrocyte in ACSF at various time points by trypan blue staining (n = 6). **(D)** Quantification of percentage of LDH release from erythrocytes in ACSF at various time points by LDH release assays (n = 6). **(E)** Optical microscope images showing the morphological characteristics of erythrocytes in ACSF at various time points (scale bars: 10 μm). **(F)** Quantification of erythrocyte size in ACSF at various time points by size analysis (n = 6). The data are presented as the means ± SEM; *P < 0.05, **p < 0.01, ***p < 0.005; ****p < 0.001; ns, not significant.

Supplemental Figure 4

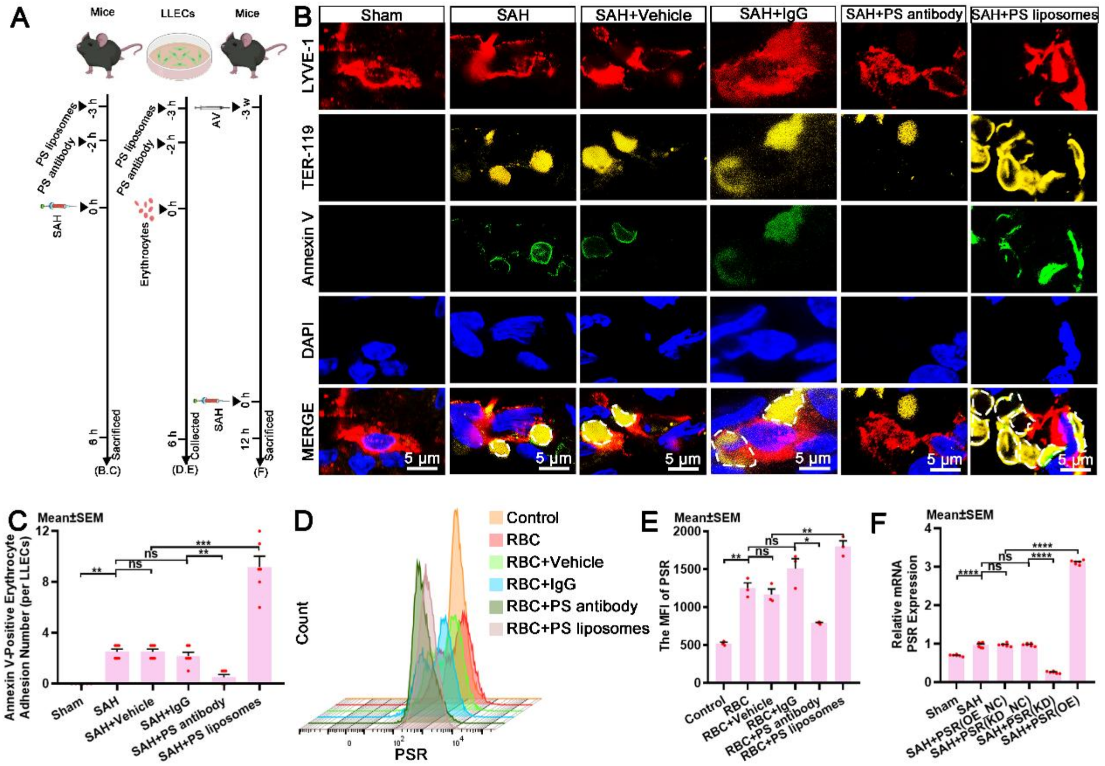

Figure S4. PS modulates the recognition of apoptotic erythrocytes by LLECs and regulates the expression of PSR. (A) Schematic of the experimental design and procedures. **(B)** Representative immunofluorescence images showing the number and adhesion status of Annexin V-positive, TER-119-positive erythrocytes and LYVE-1-positive LLECs *in vivo* (scale bars: 5 μm). **(C)** Quantification of Annexin V-positive erythrocyte adhesion number (per LLECs) by immunofluorescence staining (n = 6). **(D)** Representative flow cytometry histograms displaying the expression of PSR *in vitro*. **(E)** Quantification of the MFI of PSR by flow cytometry analysis (n = 3). **(F)** Quantification of the mRNA expression of PSR by RT-qPCR (n = 6). Annexin V, an apoptosis marker; TER-119, an erythrocyte marker; LYVE-1, a lymphatic endothelial cell marker; DAPI, a nuclear marker. The data are presented as the means ± SEM; *P < 0.05, **p < 0.01, ***p < 0.005; ****p < 0.001; ns, not significant.

Supplemental Figure 5

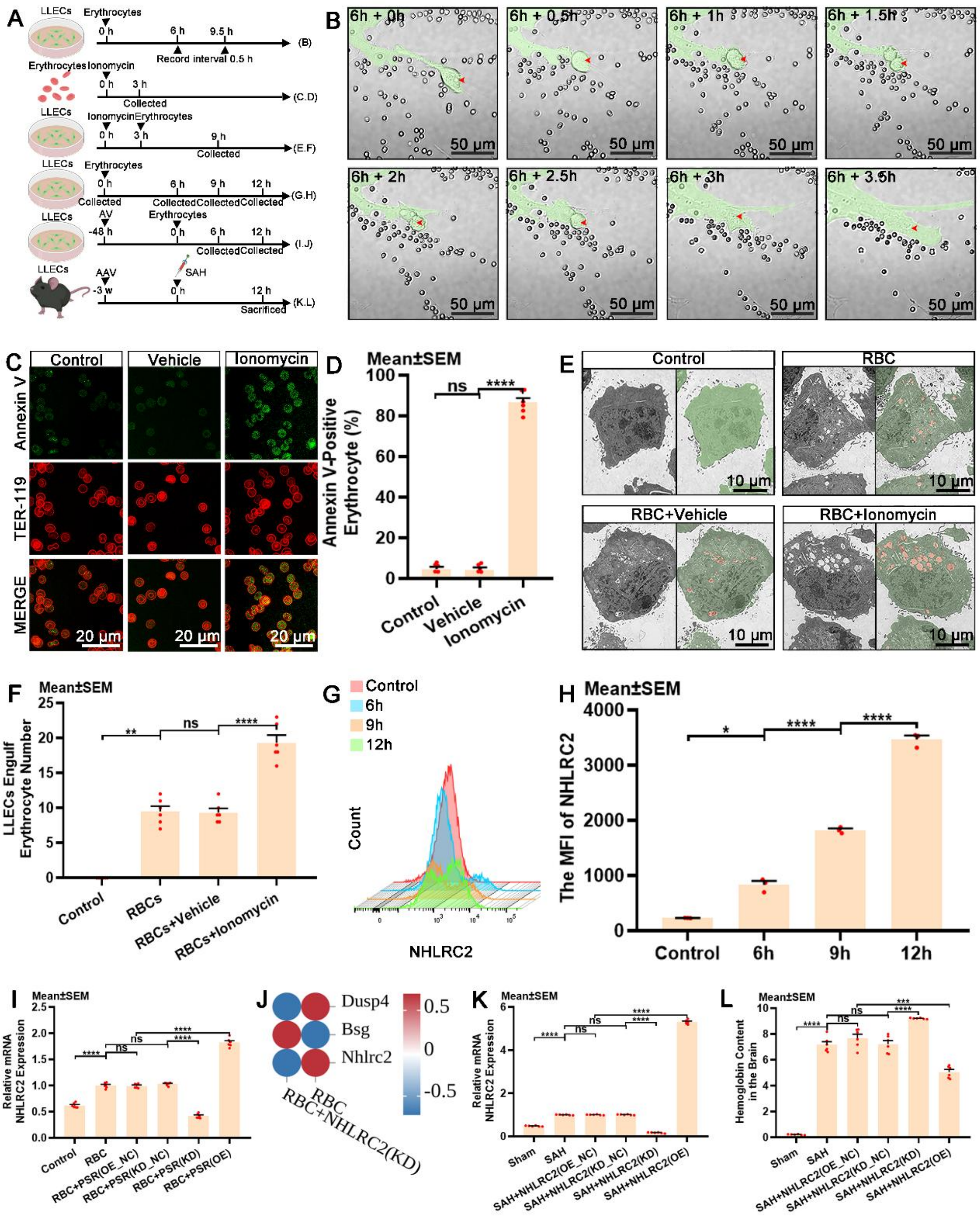

Figure S5. LLECs engulf apoptotic erythrocytes and express NHLRC2. (A) Schematic of the experimental design and procedures. **(B)** Time-lapse images documenting the dynamic process by which LLECs engulf erythrocytes (LLECs are indicated by green pseudocolor; engulfed erythrocytes are indicated by red arrows; scale bars: 50 μm). **(C)** Representative immunofluorescence images showing the expression of Annexin V-positive and TER-119-positive erythrocytes. **(D)** Quantification of the percentage of Annexin V-positive erythrocyte by immunofluorescence staining (n = 6). **(E)** Representative TEM images depicting the

engulfment interactions between LLECs and erythrocytes *in vitro* (LLECs are indicated by green pseudocolor; erythrocytes are indicated by red pseudocolor; scale bars: 10 μm). **(F)** Quantification of the number of erythrocytes LLECs engulfing by TEM (n = 6). **(G)** Representative flow cytometry histograms displaying the expression of NHLRC2 *in vitro*. **(H)** Quantification of the MFI of NHLRC2 by flow cytometry analysis (n = 3). **(I)** Quantification of the mRNA expression of NHLRC2 by RT-qPCR (n = 6). **(J)** DEGs analysis between the RBC+NHLRC2(KD) vs RBC group in efferocytosis gene set. **(K)** Quantification of the mRNA expression of NHLRC2 by RT-qPCR (n = 6). **(L)** Quantification of the hemoglobin content in the brain by hemoglobin content detection (n = 6). Annexin V, an apoptosis marker; TER-119, an erythrocyte marker. The data are presented as the means ± SEM; *$P < 0.05$, **$p < 0.01$, ***$p < 0.005$; ****$p < 0.001$; ns, not significant.

Video S1. Related to Figure S5B. Time-lapse images document the dynamic process by which LLECs engulfed erythrocytes between 6 to 9.5 hours post-co-incubation.
